# Gut and blood differ in constitutive blocks to HIV transcription, suggesting tissue-specific differences in the mechanisms that govern HIV latency

**DOI:** 10.1371/journal.ppat.1007357

**Published:** 2018-11-15

**Authors:** Sushama Telwatte, Sulggi Lee, Ma Somsouk, Hiroyu Hatano, Christopher Baker, Philipp Kaiser, Peggy Kim, Tsui-Hua Chen, Jeffrey Milush, Peter W. Hunt, Steven G. Deeks, Joseph K. Wong, Steven A. Yukl

**Affiliations:** 1 San Francisco Veterans Affairs (VA) Medical Center and University of California, San Francisco (UCSF), San Francisco, CA, United States of America; 2 Zuckerberg San Francisco General Hospital and the University of California, San Francisco (UCSF), San Francisco, CA, United States of America; Duke University Medical Center, UNITED STATES

## Abstract

Latently-infected CD4+ T cells are widely considered to be the major barrier to a cure for HIV. Much of our understanding of HIV latency comes from latency models and blood cells, but most HIV-infected cells reside in lymphoid tissues such as the gut. We hypothesized that tissue-specific environments may impact the mechanisms that govern HIV expression. To assess the degree to which different mechanisms inhibit HIV transcription in the gut and blood, we quantified HIV transcripts suggestive of transcriptional interference (U3-U5; "Read-through"), initiation (TAR), 5' elongation (R-U5-pre-Gag; "Long LTR"), distal transcription (Nef), completion (U3-polyA; "PolyA"), and multiple splicing (Tat-Rev) in matched peripheral blood mononuclear cells (PBMCs) and rectal biopsies, and matched FACS-sorted CD4+ T cells from blood and rectum, from two cohorts of ART-suppressed individuals. Like the PBMCs, rectal biopsies showed low levels of read-through transcripts (median = 23 copies/10^6^ cells) and a gradient of total (679)>elongated(75)>Nef(16)>polyadenylated (11)>multiply-spliced HIV RNAs(<1) [p<0.05 for all], demonstrating blocks to HIV transcriptional elongation, completion, and splicing. Rectal CD4+ T cells showed a similar gradient of total>polyadenylated>multiply-spliced transcripts, but the ratio of total to elongated transcripts was 6-fold lower than in blood CD4+ T cells (*P =* 0.016), suggesting less of a block to HIV transcriptional elongation in rectal CD4+ T cells. Levels of total transcripts per provirus were significantly lower in rectal biopsies compared to PBMCs (median 3.5 vs. 15.4; *P =* 0.008) and in sorted CD4+ T cells from rectum compared to blood (median 2.7 vs. 31.8; *P =* 0.016). The lower levels of HIV transcriptional initiation and of most HIV transcripts per provirus in the rectum suggest that this site may be enriched for latently-infected cells, cells in which latency is maintained by different mechanisms, or cells in a "deeper" state of latency. These are important considerations for designing therapies that aim to disrupt HIV latency in all tissue compartments.

## Author summary

Available antiretroviral drugs significantly prolong life expectancy and reduce morbidity in people living with HIV. However, HIV can escape host immune responses and drug treatment by establishing a reversibly silent ("latent") infection in CD4+ T cells. This latent infection represents the major barrier to a cure. While much of the research to date has highlighted the importance of peripheral CD4+ T cells as reservoirs for latent HIV, it is becoming increasingly apparent that the gut may play an integral role as a major tissue reservoir for HIV. In this study, we show that the transcriptional blocks that underlie HIV latency in CD4+ T cells differ in the blood and gut. In HIV-infected people on effective treatment, the major blocks to HIV transcription in blood cells occur at transcriptional elongation, distal transcription/polyadenylation (completion), and splicing. In the gut, the major block to HIV transcription occurs at transcriptional initiation, suggesting that HIV latency is maintained by different mechanisms in the gut, which may be enriched for latently-infected cells and/or cells in a "deeper" state of latency. These differences in the blocks to HIV transcription are important to consider in designing therapies that aim to cure HIV.

## Introduction

The major barrier to a cure for HIV is thought to be latently-infected cells that do not produce HIV constitutively but can be induced to produce infectious virus upon activation [1–3]. The latent HIV reservoir cannot be eliminated using currently available antiretroviral drugs, and due to their long half-lives and ability to proliferate [4], latently-infected cells can persist for many years [5–8]. While an extensive body of research has underscored the importance of peripheral CD4+ T cells as reservoirs for latent HIV, it is becoming increasingly apparent that the gut may play an integral role as a major tissue reservoir for HIV [9]. First, a large proportion of all lymphocytes reside in lymphoid tissue, of which the gut accounts for up to 85 per cent [10]. Second, CD4+ T cells of the gut are likely to be more vulnerable to infection than their peripheral blood counterparts [10]. This increased permissivity to HIV [11, 12] may be due to factors such as elevated levels of activation or CCR5 expression [13–15]. Consequently, the depletion of CD4+ T cells in the gut during acute HIV [16] and SIV [17–21] infection is both more rapid and severe than peripheral blood. Furthermore, this depletion occurs prior to and is more profound than that in the blood or lymph nodes [17, 22]. The disproportionate effect of HIV infection on the gut may result in an increased HIV burden in gastrointestinal tissue. Both HIV DNA and RNA are found to be concentrated in the gut [23, 24] and replication-competent HIV has been recovered from the rectal mucosa [25], suggesting that a proportion of gut CD4+ T cells harbor replication-competent proviruses. Prior data also suggest differences between blood and gut in infected cell types, levels of T cell activation, HIV DNA levels, relationship to activation, and levels of HIV RNA per cell [23, 26], suggesting these tissues differ in the mechanisms that govern HIV transcription and latency.

Using a novel panel of reverse transcription droplet digital polymerase chain reaction (RT-ddPCR) assays that can simultaneously quantify multiple different blocks to HIV transcription, we recently showed that the major reversible blocks to HIV transcription in peripheral CD4+ T cells from ART-suppressed patients are blocks to proximal elongation, distal transcription/polyadenylation (completion), and splicing [27]. We hypothesized that the mechanisms and degrees of HIV transcriptional blocks underlying HIV latency differ between gut and peripheral blood. In this study, we applied our "transcriptional profiling" assays to two cohorts of ART-suppressed individuals to simultaneously assess the mechanisms that govern HIV transcription in the gut and blood. We quantified the levels of different HIV RNAs in PBMCs and intact rectal biopsies (n = 9), as well as sorted CD4+ T cells from peripheral blood and dissociated rectal biopsies (n = 7). The relative levels of the different HIV RNAs suggested blocks to distal HIV transcription, completion, and splicing in all samples, and these observations were not explained by mutations in the corresponding HIV DNA primer/probe sequences or differential RNA stabilities. However, in contrast to our findings in peripheral CD4+ T cells [27], we found a much greater block to HIV transcriptional initiation in the rectum (both biopsies and sorted cells) compared to the blood. These differences in HIV transcriptional blocks, which could reflect tissue-specific differences in viral or cellular factors, are important to consider in designing therapies that aim to eliminate or silence HIV-infected cells.

## Results

### HIV transcription profile suggests successive blocks to HIV transcriptional elongation, completion, and splicing in PBMCs and rectal biopsies

**Fig 1:**
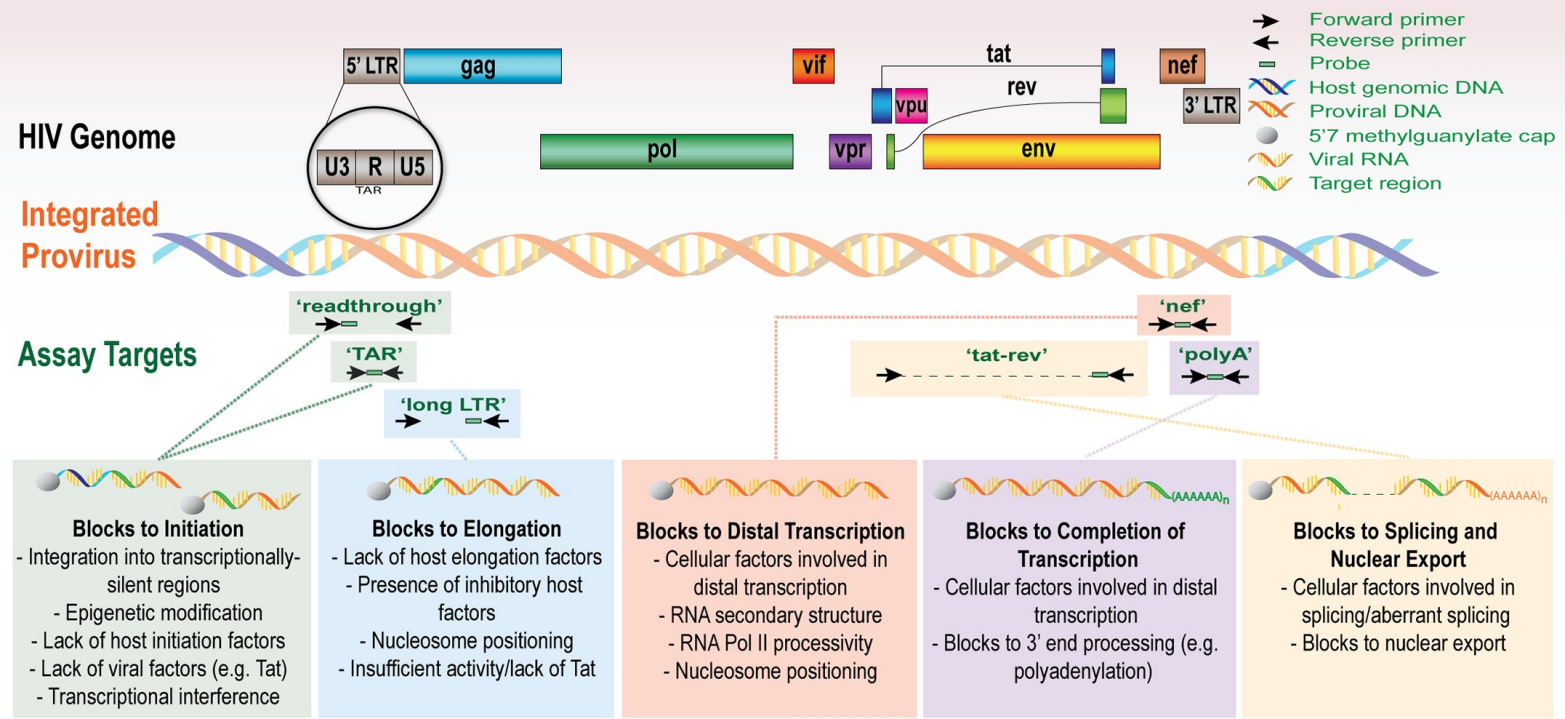
The HIV genome and the targets for transcription profiling assays. This schematic shows the genetic organization of proviral HIV DNA and the HIV ‘transcription profiling’ assays targeting specific RNA sequence regions that provide insight into blocks to transcription. Some proposed mechanisms underlying the blocks to transcription initiation, elongation, and splicing are detailed.

**Fig 2:**
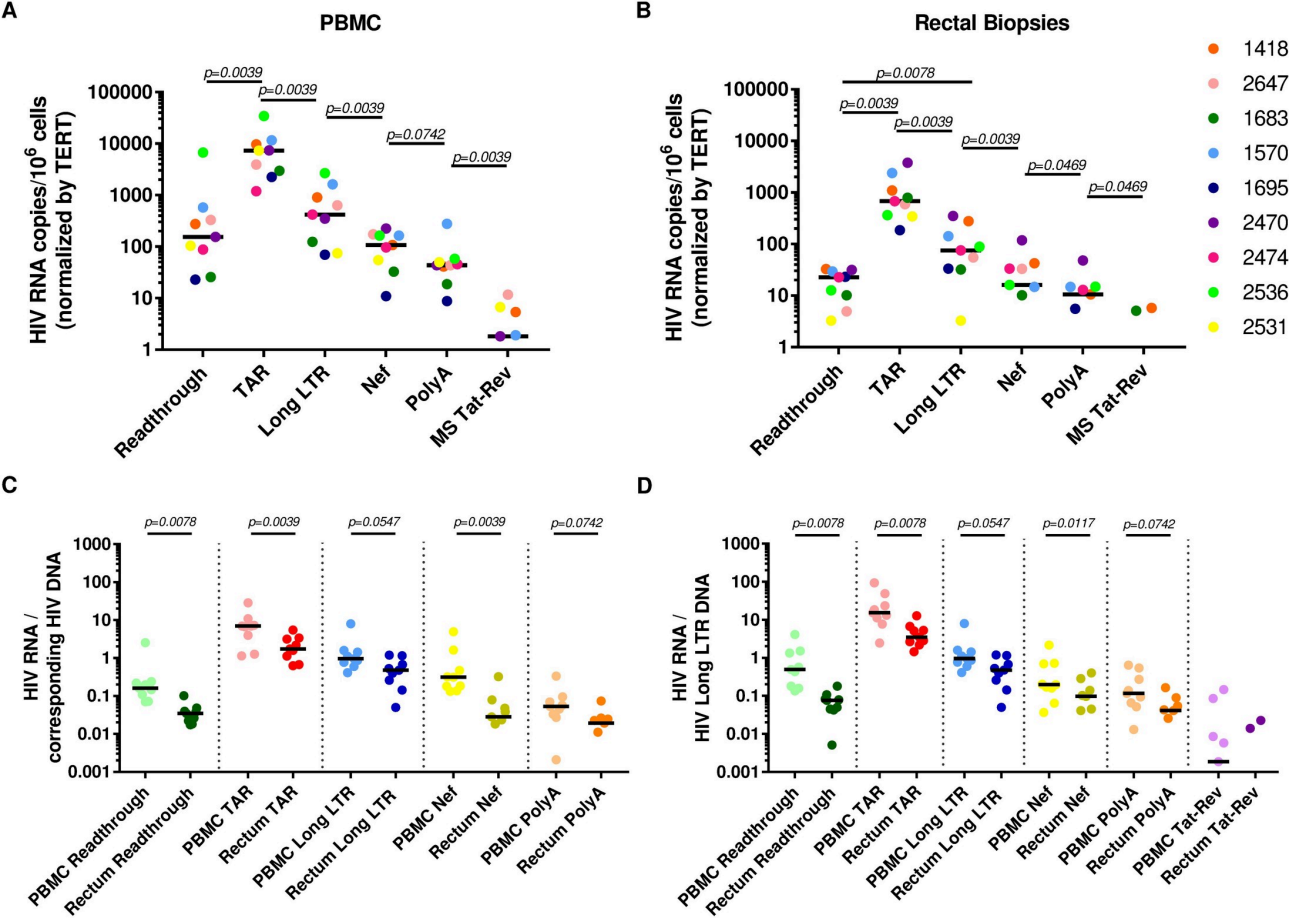
HIV RNA levels and HIV RNA/DNA ratios reveal blocks to elongation, distal transcription, and multiple-splicing in PBMCs and intact gut biopsies. Read-through, total (TAR), 5’ elongated (R-U5/pre-Gag; “Long LTR”), Nef, polyadenylated (PolyA), and multiply-spliced Tat-Rev (MS Tat-Rev) HIV RNAs were measured in (A) PBMCs; and (B) intact rectal biopsies (*n = 9* ART-suppressed individuals). 2C-D: Levels of HIV transcription per provirus are lower in the gut than the blood. Levels of each HIV RNA were normalized to HIV DNA from the same sample as measured by (C) ddPCR for the corresponding DNA sequence region (PolyA was normalized to the Read-through assay, which employs the same forward primer/probe), except for MS Tat-Rev where there is no DNA equivalent; or (D) ddPCR for the Long LTR assay, which is present once in each intact provirus. Bars indicate the median. Comparisons between transcripts were performed using the Wilcoxon signed-rank test.

We used a novel panel of HIV “transcription profiling” assays (Fig 1) to quantify HIV transcripts suggestive of transcriptional interference (U3-U5; "Read-through"), initiation (TAR [Trans-activation Response region]), 5' elongation (R-U5-pre-Gag; "Long LTR” [Long Terminal Repeat]), distal transcription (Nef), completion (U3-polyA; "PolyA"), and multiple splicing (Tat-Rev) in PBMCs and intact rectal biopsies from nine ART-suppressed individuals. In PBMCs, these assays revealed a reproducible gradient in the relative abundance of HIV transcripts (normalized to cell equivalents by ddPCR for Telomere Reverse Transcriptase [TERT]) where total (TAR) > elongated (Long LTR) > distally elongated (Nef) > polyadenylated (PolyA) > multiply-spliced Tat-Rev transcripts (medians: 7289, 420, 108, 44, and 2 copies/10^6^ cells, respectively; Fig 2A). Read-through transcripts (U3-U5), suggestive of transcriptional interference, were also detected in every individual (median 155 copies/10^6^ cells), but were 30-fold lower than total (TAR) transcripts (median [Read-through/TAR ratio] = 0.033). The median level of 5' elongated (Long-LTR) transcripts was 17-fold lower than that of total transcripts (median [Long LTR/TAR] = 0.06), suggesting a block to proximal elongation. The median level of Nef (3') was almost 4-fold lower than that of 5' elongated transcripts (median [Nef/Long LTR] = 0.26), suggesting a block to distal transcription. The median level of multiply-spliced transcripts was 23-fold lower than levels of polyadenylated transcripts (median [MS Tat-Rev/PolyA] = 0.04), in accord with prior data suggesting a reversible block to multiple splicing [27].

A similar trend in relative transcript levels (albeit at lower overall levels) was observed in the rectal biopsies, wherein the relative abundance of HIV transcripts was also: total > elongated > Nef > polyadenylated > multiply-spliced Tat-Rev (medians: 679, 75, 16, 11, and <1 copies/10^6^ cells, respectively; Fig 2B). Just as in the PBMCs, Read-through transcripts were detected in all individuals (median 23 copies/10^6^ cells), but were much lower than that of total transcripts (47-fold lower; median [Read-through/TAR] = 0.021). The median level of elongated transcripts was 9-fold lower than that of total transcripts (median [Long LTR/TAR] = 0.11). The median level of distally-elongated (Nef) transcripts was 4-fold lower than that of 5’ elongated transcripts (median [Nef/Long LTR] = 0.21), and the level of polyadenylated transcripts was nearly 2-fold lower than that of distally-elongated transcripts (median [PolyA/Nef] = 0.66]). A block to multiple-splicing is also likely (PolyA > MS Tat-Rev), given that polyadenylated HIV transcripts were detected in rectal biopsies from 6 of 9 individuals, while MS Tat-Rev transcripts were detected in biopsies from only 2 of 9 individuals (vs. 5 of 9 from PBMCs; Fig 2A and 2B). These data suggest that in both PBMCs and rectal biopsies, HIV transcription is blocked at the stages of elongation, distal transcription/polyadenylation (completion), and splicing.

### Differences in HIV RNA levels are unlikely to be driven by proviral deletions or hypermutations in primer/probe regions

To address the possibility that proviral deletions or hypermutations in primer/probe regions could account for the varying levels of HIV transcripts, we quantified the levels of U3-U5 ("Read-through"), TAR, Long LTR and Nef in DNA extracted in parallel with the RNA from the same PBMCs and rectal biopsies (*n = 9* individuals) using the same primers/probes and ddPCR conditions used to measure each HIV RNA (S1 Fig). Comparisons between DNA from the same tissue revealed no differences in the levels of TAR and Read-through regions (both present at 2 copies in an intact provirus) and these levels were ≥2-fold greater than long LTR DNA (1 copy per intact provirus) in rectal biopsies and PBMCs (median TAR/Long LTR = 1.99 and 3.49, respectively; S1 Fig). Levels of Nef DNA (1 copy per intact provirus) were similar to Long LTR DNA and tended to be lower than both Read-through and TAR DNA for both tissues (*P<0*.*05* for all comparisons).

Next, we measured the ratio of each HIV RNA to the corresponding HIV DNA sequence region quantified using the same ddPCR assay (Fig 2C) and normalized to 10^6^ cells in the same manner. This measure expresses the average level of transcription per provirus and is independent of normalization to cell numbers. For both PBMCs and intact biopsies, the gradient pattern in the levels of successive HIV transcripts was preserved after normalization of each HIV RNA region to the corresponding HIV DNA region, suggesting that the differences are unlikely to be due to proviral deletions or hypermutations in primer/probe regions.

### Lower transcription per provirus of most HIV RNAs in intact rectal biopsies compared to PBMCs

The average level per provirus of each HIV transcript was quantified using two approaches. First, we expressed the ratio of each HIV transcript to HIV DNA measured using the same primers/probe (Fig 2C), which revealed lower levels of Read-through, TAR and Nef transcripts per provirus in intact rectal biopsies compared to PBMCs (*P<0*.*05* for all). As an orthogonal method, we expressed the ratio of each HIV transcript to DNA measured using the Long LTR assay alone, which is present in only one copy per intact provirus. This analysis revealed the same trend for all transcripts (Fig 2D; *P<0*.*05* for all). Together, these data strongly suggest lower levels of HIV transcriptional initiation and distal transcription, in addition to lower levels of transcriptional interference, in the rectal biopsies relative to PBMCs.

### Differences in ex vivo HIV transcript stability do not drive the differences in levels

**Fig 3:**
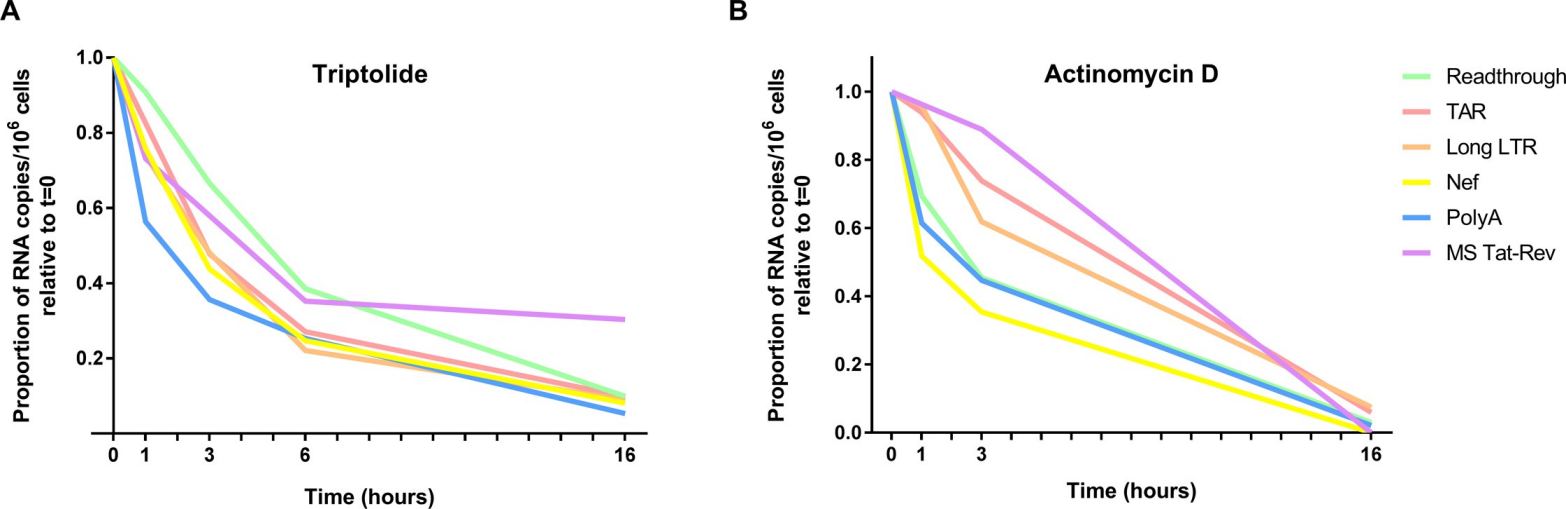
Stability of HIV transcripts *ex vivo*. Peripheral CD4+ T cells were isolated from an ART-suppressed individual and treated with the RNA polymerase II (RNA Pol II) inhibitors (A) Triptolide [100 nM] or (B) Actinomycin D [5 mg/mL] to arrest *de novo* cellular and viral transcription. HIV transcripts (Read-through, TAR, Long LTR, Nef, PolyA, and MS Tat-Rev) were quantified using RT-ddPCR from cells harvested at various time points post-treatment. Levels of each HIV RNA were expressed as a proportion of the level at time t = 0 (shown) and the half-lives were determined using a one-phase exponential decay model. Data normalized to DNA mass are shown.

To determine whether sequence-specific differences in RNA stability/degradation contribute to the divergent levels of HIV transcripts detected, we measured the RNA decay rate of each transcript in peripheral CD4+ T cells isolated from an ART-suppressed patient in the presence or absence of the RNA Pol II inhibitors Triptolide (Fig 3A) or Actinomycin D (Fig 3B). In the absence of RNA Pol II inhibitors, levels of each HIV transcript over 16 hours (h) remained relatively stable (S2 Fig). In contrast, the presence of either Triptolide (100nM) or Actinomycin D (5 mg/mL) resulted in decay of all HIV transcripts over 16h, irrespective of normalization (Fig 3, S1 Table). The half-lives of TAR- and Long LTR-containing transcripts were similar (in the order of ~3–5 hours) irrespective of treatment (Triptolide or Actinomycin D) and generally similar to the half-lives of read-through, Nef, and PolyA transcripts in the presence of Triptolide (4.58, 2.80 and 2.29h, respectively), although the latter three transcripts had shorter half lives in the presence of Actinomycin D (2.54, 1.66, and 2.34h, respectively). The half-life of MS Tat-Rev transcripts was longer with Triptolide (6.25h) and Actinomycin D (5.49h) treatments. The relatively short, similar half-lives of most HIV transcripts (S1 Table) suggest rapid decay *in vivo*, and any differences do not readily account for the measured differences in levels of the various HIV RNAs.

### HIV DNA and 2-LTR circles trend higher in rectal CD4+ T cells than blood CD4+ T cells

**Fig 4:**
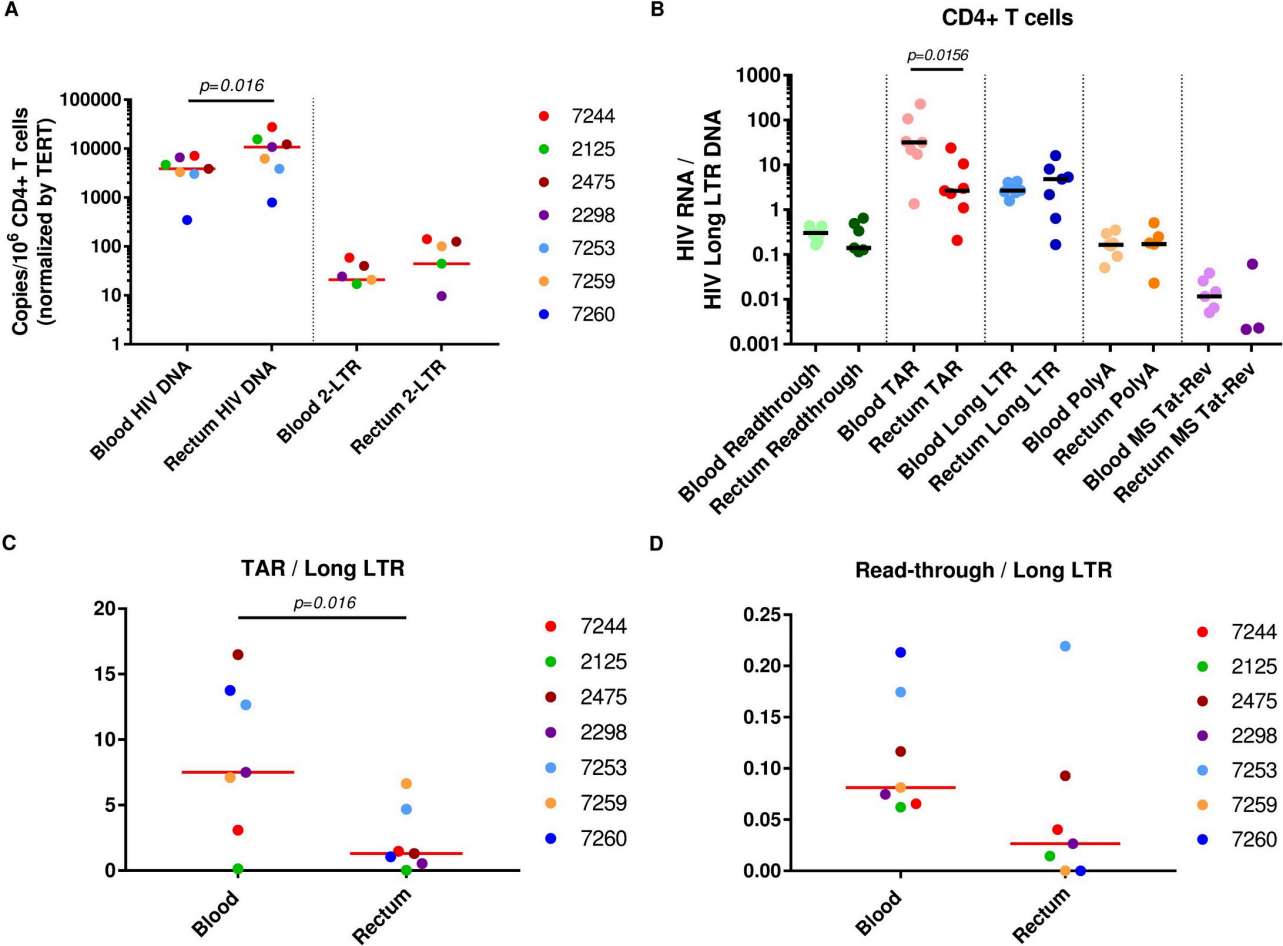
Levels of HIV DNA, 2-LTR circles, and blocks to transcription differ between CD4+ T cells from the gut and blood. (A) Levels of HIV DNA (Long LTR region) and 2-LTR circles were quantified in CD4+ T cells from the blood and rectum using ddPCR and expressed as copies per million CD4+ T cells (normalized by TERT). (B) The average levels per provirus of each transcript (ratio of each HIV RNA to the Long LTR HIV DNA) were measured in CD4+ T cells isolated from the blood and rectum *(n = 7 matched individuals)*. (C) The ratio of TAR to Long LTR RNA was determined to compare the block to transcriptional elongation in CD4+ T cells from the blood and rectum. (D) The ratio of Read-through to Long LTR RNA was determined to assess the contribution of transcriptional interference to the block to HIV transcription initiation in rectal CD4+ T cells relative to peripheral CD4+ T cells. Bars represent medians. Comparisons between transcripts were performed using the Wilcoxon signed-rank test.

Since PBMCs and gut biopsies contain different mixtures of T and non-T cells, we also compared CD4+ T cells (defined as CD3+CD8-) sorted from blood and dissociated rectal biopsies from a different cohort of seven ART-suppressed patients. HIV DNA (as measured by the Long LTR assay) was higher in CD4+ T cells from the rectum (10,736 copies/10^6^ CD4+ T cells) than blood (3,841 copies/10^6^ CD4+ T cells; *P =* 0.016; Fig 4A). HIV DNA can exist in non-integrated or episomal forms, such as 2-LTR circles, which have been interpreted as either labile markers of recent infection or stable forms that decrease only with cell division [28, 29]. Therefore, we also measured the levels of 2-LTR circles using a new assay (S3 Fig), as well as the levels of 2-LTR circles relative to total HIV DNA, in the blood and rectal CD4+ T cells by ddPCR. 2-LTR circles were detected in both blood and rectal CD4+ T cells from the same 5 of 7 individuals, and in 4 of these individuals, levels of 2-LTR circles/10^6^ CD4+ T cells (normalized by TERT) were higher in the rectum (Fig 4A). However, the ratio of 2-LTR HIV DNA to total HIV DNA did not appear to differ in CD4+ T cells from rectum and blood.

### Greater block to HIV transcription initiation in CD4+ T cells from rectum compared to blood

In order to measure blocks to HIV transcription in the sorted CD4+ T cells, levels of read-through, total, elongated, completed, and multiply-spliced Tat-Rev transcripts were measured by RT-ddPCR (S4 Fig). Since HIV DNA levels differed in the CD4+ T cells from blood and rectum, levels of each HIV RNA were divided by the HIV DNA (measured using the Long LTR assay) to express the average transcription per provirus (Fig 4B). In CD4+ T cells from both sites, we observed low average levels per provirus of read-though transcripts compared to total transcripts, suggesting little transcriptional interference, and a gradient where elongated > polyadenylated > multiply-spliced transcripts, suggesting blocks to distal transcription and splicing. For the four individuals for whom ileal CD4+ T cells were available, we observed a similar trend (S5 Fig). As in the rectal biopsies, levels of total (TAR) transcripts per provirus were much lower (median 12-fold) in rectal CD4+ T cells compared to peripheral CD4+ T cells (Fig 4B; *P = 0*.*016*), suggesting less initiation of HIV transcription in the rectum. Unlike the intact rectal biopsies, PBMCs, or blood CD4+ T cells, we observed no difference between levels of elongated (Long LTR) and total (TAR) transcripts in the rectal CD4+ T cells (Fig 4B). With the caveat that isolation of gut CD4+ T cells requires additional processing, these data suggest little or no block to elongation in the sorted rectal CD4+ T cells.

### Block to transcriptional elongation is greater in CD4+ T cells from blood compared to rectum

Ratios of one HIV transcript to another are independent of normalization to cell numbers and can be used to measure the presence and degree of different HIV transcriptional blocks [27]. We did not find a difference between PBMCs and rectal biopsies in the proportion of HIV transcripts that are read-through (read-through/total) or in the proportion of HIV transcripts that proceed through blocks to elongation (elongated/total), completion (polyadenylated/elongated), or multiple splicing (MS Tat-Rev/polyadenylated). In contrast, the sorted CD4+ T cells from blood and rectum showed a 6-fold difference in the proportion of HIV transcripts blocked at the stage of elongation (*P* = 0.016; Fig 4C), with little block to elongation in the rectal CD4+ T cells (median total/elongated = 1.31) and a strong block to elongation in CD4+ T cells from the blood (median total/elongated = 7.50). These data suggest that a block to HIV transcriptional initiation plays a greater role in inhibiting virus expression in the rectal CD4+ T cells, whereas a block to elongation likely plays a bigger role in CD4+ T cells from the blood.

### Lower transcription initiation in the gut is unlikely to be driven by transcriptional interference

Blocks to HIV transcriptional initiation could be due to transcriptional interference caused by transcription from neighboring cellular genes that perturbs assembly of preinitiation complexes at the 5’ HIV LTR [30]. To assess the likely contribution of transcriptional interference to the decreased HIV transcriptional initiation in rectal CD4+ T cells, we measured levels of read-through transcripts in relation to total and elongated HIV transcripts. A trend toward lower read-through/elongated transcripts was observed in sorted CD4+ T cells from rectum compared to blood (0.03 vs. 0.08, respectively, *P = 0*.*078*; Fig 4D), suggesting less transcriptional interference in the rectal CD4+ T cells. Furthermore, the levels of read-through transcripts tended to be low compared to total transcripts in all samples from both tissues. These findings suggest that transcriptional interference plays a relatively modest role in inhibiting HIV transcription in both sites.

### Collagenase treatment does not degrade HIV RNA or change the pattern of differences between HIV transcripts

It is conceivable that the process of dissociating gut tissue using collagenase may degrade HIV RNA, which could contribute to lower levels of HIV RNAs in the sorted gut CD4+ T cells but not the intact biopsies. Alternatively, the tissue processing could induce HIV transcriptional elongation, which could contribute to the lower block to elongation in sorted gut CD4+ T cells. To address these concerns, we treated PBMCs from an HIV-infected individual using the same protocol employed for dissociation of gut biopsies. HIV RNA transcripts were measured in PBMCs that were untreated, FACS-stained only, or collagenase treated and FACS-stained (S6 Fig). Interestingly, we found that the FACS-staining procedure necessary to sort live cells of interest may itself cause increases in HIV transcription. In PBMCs that were not treated with collagenase but were FACS-stained, an increase was observed in all HIV transcripts in relation to untreated PBMCs (S6 Fig). FACS-staining alone resulted in a 6.6- and 4.4-fold increase in MS Tat-Rev relative to untreated cells when normalized by RNA mass and TERT, respectively. In contrast, the combination of collagenase treatment and FACS-staining resulted only in an ~2.5 fold increase in MS Tat-Rev relative to untreated cells. The effect of collagenase without FACS staining was also determined in PBMCs from a second HIV-infected individual (S6 Fig). Collagenase treatment alone increased levels of all HIV RNA transcripts, although the change was less than two-fold for each transcript irrespective of normalization (S6 Fig). Collagenase treatment did not alter the pattern of differences between various HIV transcripts. Given that the flash-frozen biopsies that were not subject to collagenase treatment also demonstrated lower levels of HIV transcription, these data support our findings that RNA stability or processing disparities do not account for differences in HIV transcription between tissues.

## Discussion

The reversible lack of virus expression in latently-infected cells is widely considered to be the main barrier to cure of HIV, but it is unclear what mechanisms inhibit constitutive virus expression in lymphoid tissues such as the gut, where most infected cells reside, or whether these mechanisms differ from HIV-infected cells in the blood. In this study, we employed our "transcriptional profiling" [27, 31] approach to measure the degree to which different mechanisms inhibit HIV transcription (and hence virus expression) in gut and blood within HIV+ individuals on suppressive ART.

We measured levels of six HIV transcripts in blood and rectum from two different cohorts of ART-suppressed patients. Distinct types of samples were chosen to minimize sample processing that could affect transcription (PBMCs and flash frozen biopsies) or to facilitate comparison between the same cell type (sorted CD4+ T cells from blood and rectum). In both blood samples and the rectal biopsies, we observed a reproducible pattern in the abundance of HIV transcripts that declined in successive increments (TAR>Long LTR> Nef> PolyA > MS Tat-Rev). These findings, which accord with our previous findings in peripheral CD4+ T cells from ART-suppressed individuals [27], suggest constitutive blocks to HIV transcriptional elongation (TAR > long LTR), distal HIV transcription (Long LTR > Nef), completion (Long LTR > PolyA), and multiple splicing (PolyA > MS Tat-Rev). In contrast to these results from the blood and even the biopsies, we found little evidence for a block to elongation in the rectal CD4+ T cells. Moreover, the average levels of total (TAR) transcripts per provirus were much lower in both types of rectal samples than the corresponding blood samples, suggesting a much greater block to HIV transcriptional initiation in the rectum.

### Validations

As with any study that attempts to quantify levels of RNA or DNA, differing assay efficiencies can greatly influence the levels of RNA or DNA detected and thus interpretations of these data. Our transcription profiling approach utilizes special methods to minimize bias towards any one sequence region, and we have previously measured the performance characteristics of each assay [27, 31]. Given that all our HIV assays demonstrate similar efficiencies [27], it is unlikely that differing assay characteristics account for the marked differences between levels of the various HIV RNAs.

Internal deletions and hypermutations are present in a substantial proportion of proviral sequences [32, 33] and could cause sequence mismatches with primers or probes, which could impair detection of some HIV transcripts [33]. To assess the impact of proviral sequence, we measured the levels of each sequence region (except Tat-Rev) in the DNA from the PBMCs and rectal biopsies, and we also normalized levels of each RNA to levels of the corresponding DNA measured using the same assays employed for the HIV RNA and normalized to 10^6^ cells using the same method (Fig 2C). The gradient in levels of different HIV RNAs was preserved even after normalizing to the corresponding DNA, and was similar when all RNAs were normalized to the same Long LTR DNA region (Fig 2D), underscoring that these differences in RNA levels cannot be attributed solely to mutations in the corresponding DNA sequence regions. We were unable to quantify HIV DNA regions other than the Long LTR region in the sorted CD4+ T cells, for which the detection of 2-LTR circles consumed a large proportion of the DNA, but it seems unlikely that these would differ much from the results in PBMCs and gut biopsies.

### RNA stability

The steady state level of each HIV RNA likely reflects a balance between production (transcription) and destruction (degradation). To determine whether sequence-specific differences in RNA stability could contribute to differences in levels of the HIV RNAs, we measured the decay of Read-through, TAR, Long LTR, Nef, PolyA, and MS Tat-Rev transcripts in CD4+ T cells from an ART-suppressed individual using two RNA Pol II inhibitors, Triptolide and Actinomycin D. In Triptolide-treated cells, the half-lives of Nef (2.80h), PolyA (2.29h), and Read-through (4.58h) did not vary considerably from TAR and Long LTR (3.13h and 2.86h, respectively). MS Tat-Rev seemed to be more stable than the other HIV transcripts after treatment with Triptolide (6.25h) and Actinomycin D (5.49h). However, the very low levels of MS Tat-Rev cause considerable imprecision in calculating its half-life, and even if MS Tat-Rev transcripts were more stable, this would not explain the very low levels of MS Tat-Rev relative to other HIV RNAs.

The half-lives determined from decay of HIV transcripts in cells treated with Triptolide tended to be higher than those determined from cells treated with Actinomycin D. Triptolide acts by inducing proteasome-dependent degradation of RNA Pol II [34], whereas Actinomycin D is thought to intercalate into DNA to sterically-inhibit RNA Pol II [35]. Potential discrepancies in the half lives measured with Triptolide and Actinomycin could be due to the different mechanisms by which these agents inhibit RNA Pol II, incomplete arrest of *de novo* transcription, or imprecision due to the limited number of time points. For both Actinomycin and Triptolide, the most striking observation is that the half-lives of less abundant HIV transcripts, such as Read-through, Nef, PolyA, and MS Tat-Rev, are comparable to those transcripts that are detected at much higher levels (TAR and Long LTR). These data strongly argue that differences in RNA stability alone do not explain the differential abundance of these HIV transcripts. Although we did not have sufficient numbers of rectal CD4+ T cells to assess the stability of these transcripts in the rectum, and cellular factors may contribute to differences between cell or tissue types, it seems unlikely that there are major sequence-dependent differences between the various HIV transcripts.

Surprisingly, these data also demonstrate that the half-lives of all HIV transcripts tested were relatively short (<7 hours for all except MS Tat-Rev). The short half-life of the TAR transcripts and the average levels of TAR RNA per provirus (>2) suggest a dynamic transcriptional environment with multiple rounds of HIV transcription initiation per day per provirus in the PBMCs, in contrast to the prevailing model of transcriptionally-silent proviruses in ART-suppressed individuals. Ultimately, however, HIV transcripts may be maintained at low levels, despite their active transcription, because of rapid RNA turnover rates, particularly in activated T cells [36]. Furthermore, blocks to elongation and splicing could lead to low expression of HIV Rev, which is critical in circumventing the degradation of unspliced viral transcripts containing introns and AU-rich sequences that contribute to instability [37, 38]. The presence of instability elements within *gag* also contribute to HIV-1 unspliced mRNA instability [39]. The expression of Gag, Pol, Vif, Vpr, Vpu, and Env proteins from unspliced and partially spliced human immunodeficiency virus type 1 (HIV-1) mRNAs depends on Rev protein, and intron-containing HIV-1 transcripts undergo nuclear downregulation as they are further spliced or degraded in the absence of Rev [39, 40]. This finding may contribute to the short half-lives of unspliced or partially spliced transcripts *ex vivo*, even those that have been polyadenylated.

Our data from collagenase-treated PBMCs provide evidence that collagenase treatment of gut cells is unlikely to promote HIV transcript degradation or selectively alter levels of a particular transcript. Collectively, our RNA stability and collagenase treatment data suggest that the differential expression of HIV transcripts in the gut and blood are not explained by either intrinsic or treatment-mediated changes to HIV RNA stabilities.

### Larger block to HIV transcriptional initiation in the rectum

Although the PBMCs and rectal biopsies differ in cell composition, direct comparison between HIV transcription in these samples was possible using the ratio of HIV RNA to HIV DNA, which yields a measure of average transcription per provirus. In intact rectal biopsies, the average levels per provirus of Read-through, total, elongated, Nef, polyadenylated, and multiply-spliced Tat-Rev transcripts were all lower compared to PBMCs, supporting previous work that reported lower levels of HIV transcription in the rectum [23, 26]. The sorted CD4+ T cells showed largely congruent results, where the average levels per provirus of Read-through, total, and multiply-spliced Tat-Rev transcripts were all lower in the rectal CD4+ T cells than the blood CD4+ T cells. One notable exception was the average level per provirus of elongated transcripts, which did not appear to differ between rectal and peripheral CD4+ T cells. Moreover, the TAR/Long LTR ratios suggest a considerable block to elongation in the peripheral CD4+ T cells (TAR/Long LTR = 7.5) but little block to elongation in the rectal CD4+ T cells (TAR/Long LTR = 1.31).

This lack of a block to elongation in the rectal CD4+ T cells seems to disagree with the results from the intact biopsies, where we observed a larger block to elongation. Aside from differences in the patient cohorts, it is likely that the rectal biopsies contain a different mix of infected cell types, including non-T cells. Alternatively, it is possible that the tissue processing (collagenase digestion and shearing) used to dissociate the rectal biopsies into single cells could change cellular or viral transcription in ways that selectively induce elongation. The control experiments in PBMCs suggest that collagenase and FACS staining may cause a small increase in all HIV transcripts, likely reflecting an increase in initiation rather than a specific effect on elongation, although it is possible that these effects of tissue processing differ in adherent and nonadherent cells. However, other findings in the sorted gut CD4+ T cells accord with those in the flash-frozen gut biopsies (which were not subject to such processing) and suggest that blocks to HIV transcriptional initiation (low TAR RNA per provirus) and blocks to later stages of HIV transcription (distal transcription, completion, and splicing) may be important for HIV latency in the gut.

**Fig 5:**
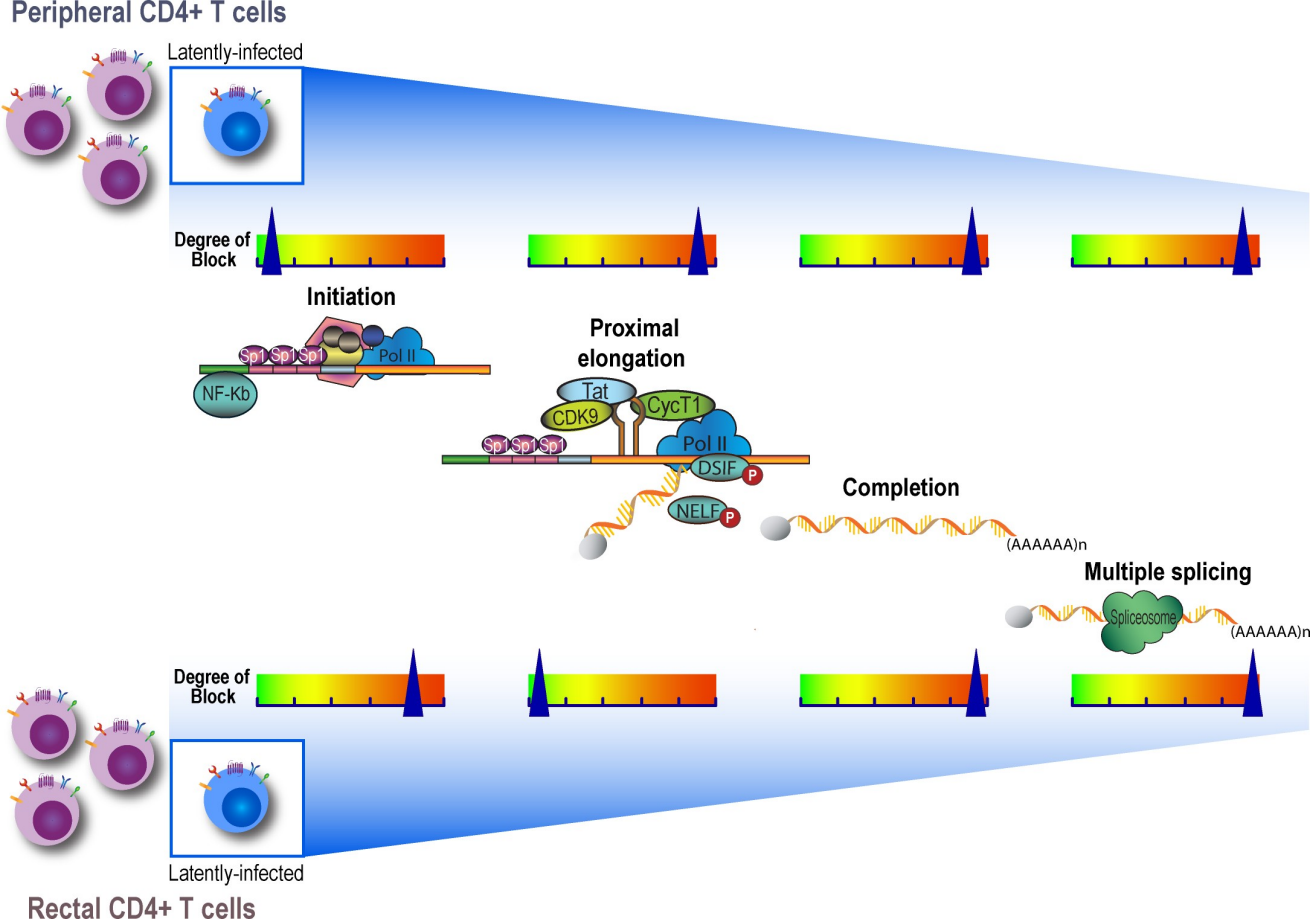
Blocks to HIV transcription differ in the gut and blood. Multiple blocks to HIV transcription occur *in* vivo and these differ in CD4+ T cells from the rectum and peripheral blood. Selected examples of potential factors are shown.

Our major finding that HIV transcription initiation in the rectal CD4+ T cells is 12-fold lower than that observed in blood CD4+ T cells (Fig 4B) provides evidence that differing transcriptional blocks operate in different tissues (Fig 5). These findings are particularly striking given that the gut harbors a much higher proportion of activated CD4+ T cells, and that T cell activation usually stimulates HIV transcription and reverses latency [10]. It is not clear what mediates the 12-fold greater block to initiation of HIV transcription in the rectum. Blocks to HIV transcriptional initiation have been attributed to integration into heterochromatin, epigenetic modification, transcriptional interference, lack of host transcription initiation factors, or insufficient activity of the viral transcription factor Tat [41–44]. It seems very unlikely that the greater block to HIV transcriptional initiation in the gut is driven by higher levels of transcriptional interference from neighboring cellular genes, since levels of Read-through transcripts per provirus tended to be lower in the gut than blood, the ratio of Read-through to elongated transcripts tended to be lower in rectal CD4+ T cells than the blood CD4+ T cells, and T cell activation is supposed to reverse transcriptional interference.

The differences between the blood and gut in the blocks to HIV transcription could be attributable to either the characteristics of the proviral sequences found in each site or the prevailing host cellular conditions and/or cellular environment. It is possible that proviruses in the rectum are more likely to be integrated into transcriptionally-silent regions, although most studies from the blood suggest that HIV is more likely to be integrated in actively-transcribed genes [45, 46], and the higher levels of T cell activation in the gut might also correlate with more actively transcribed genes. It is also possible that proviruses in the gut have more or different mutations that could affect transcription, such as those in the LTRs, Tat, or Rev [47–49]. Compartmentalization of HIV could occur in anatomic sites or tissues where viral trafficking may be impaired or restricted, such as the brain, central nervous system and genital tract [50–52], but prior studies disagree on whether there is compartmentalization of HIV in the gut [47–49]. The HIV DNA levels from the rectal biopsies suggest that many proviruses in the rectum have 2 full LTRs (containing U3-U5 and TAR) for every R-U5/pre-Gag and Nef region, but future studies will be needed to determine whether HIV integration sites or full-length proviral sequences differ between blood and gut.

Epigenetic modifications, host cell factors, and extracellular milieu at either site may also dictate the basal transcriptional activity and the consequences for silencing integrated HIV [30, 45, 46, 53–57]. Previous reports have shown that CD4+ T cell tolerance and anergy can be mediated by epigenetic modification [58–60]. Given that epigenetic modification (for example, of the LTR) can also inhibit HIV transcription [56], it is possible that the unique environment of the gut favors both induction of CD4+ T cell tolerance and HIV latency through epigenetic modification. The gut and blood also show differences in the phenotypes of HIV-infected and uninfected cells, which likely differ in the cellular factors that govern HIV transcription. Naïve and central memory T helper (T_CM_) cells account for the largest proportion of T lymphocytes in peripheral blood, while effector memory (T_EM_) and transitional memory (T_TM_) constitute the predominant populations in the gut [10]. Most HIV DNA in the blood is found in central and transitional memory CD4+ T cells [4], whereas in the gut, most HIV DNA and RNA are found in effector memory CD4+ T cells [26]. The expansive surface area of the GI mucosa is under constant exposure to diverse microbial and food antigens, resulting in sustained immune activation [10]. A higher proportion of gut CD4+ T cells express HIV coreceptors and markers of T cell activation, which may enhance their susceptibility to infection or depletion during acute infection [10]. The gut is also enriched for tissue resident memory cells and different subsets of helper T cells, such T_FH_ [61–64], TH_17_, TH_1_/TH_17_ and TH_22,_ which may serve as reservoirs for latent HIV [10]. In addition, a higher proportion of gut CD4+ T cells express immune checkpoint blockers such as PD-1 and CTLA-4 [65, 66], which have been associated with HIV latency [66]. Non-T cells such as macrophages or dendritic cells might also constitute a larger portion of the infected cells in the gut [26]. These differences in infected cell types likely contribute to the discordant progression through various transcriptional blocks observed in these two tissues.

It is not clear which cellular or viral factors might explain the differences between HIV transcriptional blocks in the blood and gut. A multitude of cellular factors reportedly interact with the HIV LTR to control transcription, and a regulatory feedback mechanism mediated by Tat and Rev drives HIV transcription through its distinct phases [67, 68]. MS Tat-Rev was more frequently detected in the blood than the gut, resulting in a lower median level per provirus in the gut, but MS Tat-Rev may have been harder to detect in the gut because of the lower frequency of CD4+ T cells in the rectal biopsies and the lower yield of sorted CD4+ T cells from the rectum. If the lower block to elongation in the rectal CD4+ T cells is not the result of induction of elongation during collagenase digestion, it could reflect higher levels of P-TEFb or lower levels of NELF in the gut CD4+ T cells, which are more likely to be activated. At the same time, activation should also increase cellular factors that increase HIV transcriptional initiation, such as NFAT and NF-κB, but HIV transcriptional initiation was lower in the rectum, even in the flash frozen biopsies. It is possible that "activation" markers have different meanings or that T cell activation results in different changes in the gut, where immune cells are exposed to more microbial products but mechanisms are needed to maintain tolerance to the normal flora.

In both blood and rectum, we found evidence suggesting blocks to HIV transcriptional completion and multiple splicing. The block to completion could represent incomplete processivity of the RNA polymerase II, which could be modulated by protein levels or post-translational modifications of the enzyme itself, cellular co-factors that affect transcriptional processivity, or secondary structure in the HIV DNA or RNA. In addition, the block to completion could reflect viral and cellular factors involved in end processing of HIV transcripts, including Vpr [69], CDK11 [70, 71], and members of the polyadenylation complex. We have previously shown that the block to multiple splicing in blood CD4+ T cells is partially reversed by T cell activation, which could change levels of cellular factors involved in splicing, such as spliceosome components, SR proteins, MATR3, and PSF [67]. However, a difference between polyA and Tat-Rev remained even after activation, which could reflect intrinsic sequence-dependent factors that inhibit HIV splicing (including multiple inefficient splice donor and acceptor sites, intronic and exonic splice silencers, secondary structure, etc. [67]) as well as cell-specific differences in activation, proviral mutations affecting Tat-Rev or splice sites [32, 33], and the effect of Rev to export unspliced HIV RNA [67].

The blocks to splicing found in both gut and blood suggest that latency could be governed, in part, by post-transcriptional mechanisms. This assertion is supported by the observation that the various latency-reversing agents tested to date fail to completely eradicate infected cells despite inducing viral transcription, albeit to varying degrees [27, 72]. Post-transcriptional blocks to HIV expression, such as blocks to nuclear export [73], RNA interference [74–76], and inefficient translation [77], have been observed in latency models and patient cells. In a primary cell model of latency, levels of intracellular Gag protein were found to be markedly low despite high levels of gag RNA [77], and in resting CD4+ T cells from ART-suppressed individuals, both partially- and fully-spliced HIV transcripts were retained in the nucleus [73], alluding to a block to nuclear export of HIV RNAs. Polyadenylation is important for nuclear export [38] as well as translation, so the block to completion could contribute to lack of export or translation. Because Rev protein facilitates export of unspliced and incompletely-spliced HIV transcripts from the nucleus [67], the block to multiple splicing could also contribute to very low levels of Rev and therefore blocks to nuclear export of unspliced and incompletely-spliced HIV transcripts. Our methods do not allow us to detect other post-transcriptional blocks, which are possible but would have to be in addition to the blocks described here.

Additional studies are needed to determine what cellular factors govern the different blocks to HIV transcription and how cellular gene expression differs between infected and uninfected cells in the blood and gut. Although a limited number of gut biopsies and cells can be obtained from endoscopic procedures, gut cells from HIV-infected patients could be tested *ex vivo* for their responses to T cell activation or drugs known to act on distinct cellular factors, and gut biopsies could be obtained during clinical trials with interventions designed to disrupt latency. Other techniques of gut cell isolation, such as those that require either no or alternative collagenases [78, 79], could be explored in an effort to minimize tissue processing and potentially increase dissociated cell recovery. Even if cell recovery is limited, single cell transcriptomic and/or proteomic studies could also be used to investigate the cellular factors that are associated with different HIV transcripts in blood and gut cells from patients. Moreover, larger cell numbers could be obtained from unused tissue left over from surgical resections in either HIV+ or HIV- patients. Future mechanistic studies could also employ primary cell latency models derived from lymphoid tissue, such as ‘human lymphocyte aggregate culture’ [80] or ‘lamina propria aggregate culture’ [81] systems, which might achieve higher numbers of HIV-infected cells.

### Limitations

In addition to the points addressed above, other limitations of this study should be noted. One limitation is the relatively small number of ART-suppressed individuals in each of the two cohorts from whom we had samples from blood and gut. Nonetheless, given the statistically significant findings from two different cohorts with two distinct types of gut samples, and findings that largely concur with previous work [23, 27], it is likely that there are differences between blood and gut in the molecular mechanisms that constitutively block HIV transcription. A second limitation is that we did not address the degree to which these blocks are reversible after T cell activation. However, we have previously demonstrated the reversibility of the blocks to HIV transcriptional elongation, completion, and splicing in blood CD4+ T cells [27], so we also expect some reversibility in gut cells, although the magnitude may differ. Finally, although our transcriptional profiling technique enables us to simultaneously assess multiple blocks to transcription, these assays are unable to distinguish which of these transcripts originate from replication-competent proviruses. Given the many cellular factors that can influence HIV transcription independently of viral fitness, the large proportion of the HIV genome in which mutations could eliminate infectivity without affecting transcription (or, conversely, could prevent any transcription), and prior evidence suggesting these blocks to HIV transcription operate in most infected CD4+ T cells from the blood, it seems likely that the same mechanisms operate in many cells with infectious proviruses. However, this question is extremely important and should be addressed in future studies, although it is an exceedingly challenging question to answer using patient cells and will require novel single cell approaches.

### Implications

Findings from this study have important implications. The large disparities between levels of different HIV RNAs in both blood and gut highlight the importance of critically evaluating the regions targeted when quantifying HIV RNA in different tissue compartments. This fact is particularly important when designing studies to evaluate the effectiveness of interventions designed to reverse latency, since putative "latency reversing" agents (LRAs) exert differential effects on the various transcriptional blocks in blood CD4+ T cells [27], and the same is likely true in other tissues. For such studies, a multi-target approach, such as the transcriptional profiling technique applied in this study, might yield greater insight than quantifying unspliced HIV RNA alone.

The lower levels of HIV transcriptional initiation and of most HIV transcripts per provirus in the rectum suggest that this site may be enriched for latently-infected cells, cells in a "deeper" state of latency, or cells in which latency is maintained by different mechanisms. Future studies are needed to determine whether the same finding holds true in other gut regions or tissues, and whether these tissues differ from blood in the proportion of cells that contain infectious proviruses or can be induced by activation to produce infectious viruses. Since infected cells in the rectum make less HIV RNA (and likely less HIV protein) [23], they may be less likely to trigger cell-intrinsic defense mechanisms or extrinsic immune responses that are designed to recognize and kill infected cells. This may facilitate survival of infected cells in the rectum and might contribute to the higher levels of HIV DNA per million CD4+ T cells in the gut. For the same reason, HIV-infected cells in the rectum might be less susceptible to killing by immune-based therapies that require HIV protein or antigen expression, such as broadly-neutralizing antibodies to Env, immunomodulators, CAR T cells, and vaccines designed to elicit B or T cell responses.

Given the stronger block to HIV transcriptional initiation, as well as persistent blocks to later stages of HIV transcription (completion, splicing) despite higher levels of T cell activation, infected cells in the rectum may also be less susceptible to agents designed to reverse latency or may require different LRAs or combinations. Direct evidence for this idea comes from a recent multi-dose trial of vorinostat in HIV-infected patients, which showed that the median increase in cell-associated HIV RNA in the rectum was 5-fold less than in the blood [82]. Future studies should investigate how gut cells differ from blood in the response to different LRAs, and whenever feasible, the gut should probably be sampled in clinical trials designed to evaluate new therapies aimed at HIV cure. Additional studies should investigate how cellular gene expression differs between CD4+ T cells in the gut and blood, since differentially-expressed genes might suggest new proteins or pathways involved in suppressing HIV transcription. As an alternative to latency reversal, some recent studies have investigated therapies designed to create a "deeper" latency and prevent reactivation from latency [83–85]. Infected gut cells that are activated *in vivo* but do not transcribe RNA could serve as a model for therapies designed to silence HIV-infected cells. In these ways, an improved understanding of the mechanisms that govern HIV transcription/latency in the gut and blood could help inform new therapies aimed at HIV cure, functional cure, or reducing HIV-associated immune activation and organ damage.

## Materials and methods

### Ethics statement

The study was approved by the Committee on Human Research (CHR), the Institutional Review Board for the University of California, San Francisco (approval #11–07551). All study participants provided written informed consent.

### Study participants

The study participants were HIV-infected adults on suppressive ART from two cohorts (median age = 51; median CD4 count = 611 cells/mm^3^; median years of suppression = 5). Matched rectosigmoid biopsies and cryopreserved PBMCs were obtained from study participants (Table 1) in the Reservoirs and Drug Levels (RADL) study. This study was a prospective, randomized study designed to measure HIV levels and antiretroviral drug (ARV) levels after suppression of plasma viremia with ART regimens containing 2 nucleotide inhibitors of reverse transcriptase and either an integrase inhibitor or a protease inhibitor. Inclusion criteria included: confirmed HIV-1 infection, ART suppression for ≥12 months on initial ART regimen, plasma HIV-1 RNA <40 copies/mL, and a willingness to undergo rectal biopsy. Matched blood and gut biopsies were obtained and aliquoted for parallel measurement of ARV levels and HIV levels. Six rectosigmoid biopsies (flash frozen) and 10^7^ cryopreserved PBMCs were available from 9 study participants for measurement of HIV levels. Ileal biopsies were available for three of the 9 study participants.

While these samples offer the advantages of an inclusive mix of cell types and less processing that could affect HIV RNA or drug levels, the PBMCs and gut biopsies also differ in composition of infected and uninfected cells. For more direct comparison of CD4+ T cells in the two tissues, we also sorted CD4+ T cells (defined as CD3+CD8-) from fresh gut biopsies (rectosigmoid +/- ileum) and blood from a different group of 7 ART-suppressed study participants recruited prospectively and sequentially from the SCOPE cohort (Table 1).

**Table 1:** Characteristics of HIV-infected study participants.

Participant ID	Cohort	Gender	Age	CD4 (cells/mm^3^)	VL	Drug Regimen	Years of suppression
2125	SCOPE	Male	48	828	<40	FTC/TDF, DRV, RTV	10
2298	SCOPE	Male	63	254	<40	FTC/TDF, ATV, RTV	9.1
2475	SCOPE	Male	54	438	<40	FTC/TDF, ATV, RTV, RAL	5.3
7253	SCOPE	Male	62	335	<40	FTC/TDF/EFV	5.5
7259	SCOPE	Male	57	523	<40	FTC/TDF/RPV	Not available
7260	SCOPE	Male	56	388	<40	ABC/DTG/3TC	Not available
7244	SCOPE	Male	52	432	<40	FTC/TDF, RAL	10
1418	RADL	Male	44	717	<40	FTC/TDF, ATV, RTV	4.9
1570	RADL	Male	54	592	<40	FTC/TDF, ATV, RTV	2.9
1683	RADL	Male	44	837	<40	FTC/TDF, DRV, RTV	3.9
1695	RADL	Female	38	688	<40	FTC/TDF, ATV, RTV	5
2470	RADL	Male	47	773	<40	FTC/TDF, ATV, RTV	6.4
2474	RADL	Male	53	630	<40	FTC/TDF, ATV, RTV	4.6
2531	RADL	Male	50	1850	<40	FTC/TDF, RAL	3.4
2536	RADL	Male	40	913	<40	FTC/TDF, RAL	2.3
2647	RADL	Male	32	587	<40	FTC/TDF, DRV, RTV	3.3

Abbreviations: VL = viral load; 3TC = lamivudine; ATV = atazanavir; DRV = darunavir; DTG = dolutegravir; EFV = efavirenz; FTC = emtricitabine; RAL = raltegravir; RPV = rilpivirine; RTV = ritonavir; TDF = tenofovir

### HIV transcription profile in PBMCs and rectal biopsies

For flash frozen rectosigmoid tissue, 1 mL TRI Reagent with 2.5 μL polyacryl carrier (both from Molecular Research Center, Cincinnati, OH) was added to the pooled rectal biopsies (six rectal biopsies per individual), which were homogenized using a Mini Beadbeater (Biospec Products, Bartlesville, OK). PBMCs from the same participants were thawed quickly and pelleted by centrifugation (300*g* for 5 min at 4°C). Following centrifugation, cryopreservation medium was removed and 1 mL TRI Reagent with 2.5 μL polyacryl carrier was added to homogenize cells. Total cellular RNA and DNA were subsequently extracted per the TRI Reagent protocol with back extraction for DNA[27].

RNA and DNA concentrations and quality were measured using the Nanodrop 1000 spectrophotometer (Thermo Fisher Scientific, Waltham, MA). Up to 1μg of total RNA was used for a polyadenylation-reverse transcription-ddPCR assay for the TAR region, which is found in all HIV transcripts. This assay employs an initial polyadenylation step, since efficient reverse transcription (RT) of short, prematurely-terminated TAR transcripts requires RT from a linker molecule [31]. Following an RT reaction employing a combination of oligo (dT) and random hexamers, replicate 5μL aliquots of the cDNA were used in a ddPCR reaction containing TAR-specific primers and probe. Up to 5μg of RNA was used for a separate 50μL common RT reaction, from which aliquots of cDNA (5μL/well) were used in ddPCR assays for other sequence regions, including U3-U5 ("Read-through"), R-U5/pre-Gag ("Long LTR"), Nef, U3-R-polyA ("Poly A"), and multiply-spliced Tat-Rev (MS Tat-Rev) regions [27].

Since prior studies suggest that the average level per cell of "housekeeping" transcripts and the average RNA content per cell differ between cell types or even between corresponding cell types in different tissues or conditions (such as T cell activation [27, 86, 87]), we did not use housekeeping transcripts or total cellular RNA to determine the cell equivalents in the RNA from the PBMCs or rectal biopsies, which contain different mixtures of cell types from different tissues. Instead, we determined the total cell equivalents in the DNA extracted from the same samples as the RNA by measuring the absolute copy numbers of a nonduplicated cellular gene, Telomere Reverse Transcriptase (TERT), using ddPCR [88]. Using the assumption that the extraction efficiency is similar for both DNA and RNA, which we have verified in prior TRI reagent extractions from PBMCs, we normalized the absolute copy numbers of cDNA in each well to copies per million cells by using the total cell equivalents in the DNA and the fraction of the total RNA used for each RT and ddPCR [27]. It should be noted that the same method of normalization was used for each HIV RNA transcript, and therefore would not explain any difference between levels of various HIV RNAs in the same sample. As an additional method of normalization, we also normalized levels of each HIV RNA to the total cell equivalents in the DNA as determined by DNA mass, using the concentration measured by NanoDrop and the total resuspension volume. The findings were essentially identical.

We also quantified the levels of the U3-U5 ("Read-through"), TAR, Long LTR, and Nef regions in DNA extracted in parallel with the RNA from the same PBMCs and rectal biopsies (from *n = 9* individuals) using the same primers/probes and ddPCR conditions used to measure each HIV RNA [27]. Since the PolyA assay does not detect HIV DNA, polyadenylated transcripts were normalized to HIV DNA levels of the U3-U5 (Read-through) assay, which contains the U3-R region of the PolyA assay and shares the same forward primer and probe. Note that we excluded MS Tat-Rev from this analysis, since this assay also does not detect HIV DNA and spans the two exons of *tat* and *rev*, so there is no DNA equivalent. Cellular DNA was fragmented by passage through a QIAshredder column [88]. Levels of each HIV DNA sequence region were measured by ddPCR in replicate aliquots (at least 2) of 500ng DNA/well and expressed as copies per million cells by levels of TERT (measured in duplicate) in other aliquots from the same DNA.

To further assess the effect of proviral sequences, we calculated the ratio of each HIV RNA to the corresponding HIV DNA sequence region quantified using the same ddPCR assay and normalized to 10^6^ cells in the same fashion. This measure expresses the average level of transcription per provirus and is independent of normalization to cell numbers. While this measure may be best to assess the impact of proviral mutations in each sequence region, it should be noted that some sequence regions (TAR, "Read-through") are present in 2 copies per intact HIV DNA (one in each LTR), while the others are present in only one copy. As an alternative method to express the average transcription per provirus in the PBMCs and biopsies, we also calculated the ratio of each HIV RNA to HIV DNA as measured using the R-U5/pre-Gag (Long LTR) assay, which is present in only one copy per intact provirus. This method also allows comparison of Tat-Rev to the other transcripts. Finally, we calculated the ratios of one HIV transcript to another, which are independent of normalization to cell numbers and can be used to measure the presence and degree of different HIV transcriptional blocks [27].

### RNA stability

To determine whether sequence-specific differences in RNA stability could contribute to differences in levels of the HIV RNAs, we measured the decay of Read-through, TAR, Long LTR, Nef, PolyA, and MS Tat-Rev transcripts in CD4+ T cells from an ART-suppressed individual using RNA Pol II inhibitors, Triptolide and Actinomycin D. CD4+ T cells were isolated from blood from an ART-suppressed individual using the Dynabeads Untouched Human CD4 T cells kit (Thermo Fisher, Waltham, MA). Replicate aliquots of CD4+ T cells (6x10^6^ cells/well) were seeded into 6-well tissue culture plates (Corning Inc., Corning, NY) at a concentration of 1x10^6^ cells/ml in complete RPMI with either DMSO (negative control), 100nM Triptolide (Sigma, St Louis, MO), or 5mg/ml Actinomycin D (Sigma, St Louis, MO). Cells were harvested at the following time points: DMSO: 0, 1 and 16h; Triptolide: 0, 1, 3, 6 and 16h; Actinomycin D: 0, 1, 3 and 16h. HIV transcripts (Read-through, TAR, Long LTR, Nef, Poly A, and MS Tat-Rev) were quantified using RT-ddPCR as described above. Levels of each HIV RNA were quantified by RT-ddPCR, normalized by alternative measures (cell counts, DNA mass, RNA mass), and expressed as a fraction of the value at time zero. The half-life for each transcript was determined using an exponential one-phase decay model.

### HIV DNA levels and transcription profile in sorted CD4+ T cells from blood and rectum

For more direct comparison of CD4+ T cells in the blood and gut, we also analyzed CD4+ T cells isolated from blood and rectosigmoid (+/- ileum) from a different group of 7 ART-suppressed individuals. Fresh gut biopsies (15–20) were obtained by colonoscopy, placed immediately in RPMI (supplemented with L-Glutamine, penicillin, streptomycin and 15% fetal calf serum), washed, and dissociated into total gut cells using collagenase with DNase and mechanical shearing [26]. Blood was obtained immediately before colonoscopy and PBMCs were isolated using Ficoll as previously described [26]. PBMCs and total gut cells were counted, stained with LIVE/DEAD stain and fluorescently-conjugated antibodies, and sorted for live, single, CD45+CD3+CD8- cells as previously described [89]. Sort yields from the rectum ranged from 83,474 to 976,738 (median 360,279; S2 Table). Cells were sorted into FACS buffer, centrifuged to pellet cells, and immediately frozen.

Total cellular RNA and DNA were isolated from the sorted CD3+CD8- cells using TRI Reagent, as described previously [26]. DNA was resuspended in 20μL (for rectal T cells) to 75μL (for blood T cells with higher cell counts) of QIAGEN buffer EB. 2.2μL were used to measure the DNA cell equivalents by ddPCR for TERT in duplicate (1μL/well). At least one (and up to 3) aliquots (5μL/well) were used to measure HIV DNA by ddPCR for the "Long LTR" region. At least two (and up to 4) aliquots (5μL/well) were used to measure levels of HIV DNA 2-LTR circles using a new ddPCR assay (see below). Levels of HIV DNA (Long LTR) and 2-LTR circles were normalized to copies/10^6^ cells using the absolute levels of TERT (2 copies/cell) in the DNA and the volumes used for each assay.

Total cellular RNA was resuspended in 20μL of RNase-free water. 5–10μL (no more than 1μg) was used for the polyadenylation-RT-ddPCR assay for TAR, while the remainder was used for a common RT reaction from which aliquots were used in replicate ddPCR assays for Read-through, Long LTR, PolyA, and MS Tat-Rev transcripts (S4 Fig) [27]. Since the average level per cell of "housekeeping" transcripts may differ in CD4+ T cells from blood and gut (which consist of different mixtures of T_H_ subtypes with differing proportions of activated cells), and we wanted to reserve as much of the RNA as possible for measurement of the 5 different HIV transcripts, we did not attempt to measure levels of housekeeping transcripts. Instead, HIV RNA levels were normalized to copies per million cells using the total cell equivalents in the DNA, as measured using ddPCR for TERT. As an alternative method to normalize the HIV RNA levels to cell numbers, we also used total cell counts from the sorts; findings were the same.

To express the average level of each transcription per provirus in the sorted CD4+ T cells, we calculated the ratio of each HIV RNA to HIV DNA (both expressed as copies/10^6^ cells using normalization to TERT levels) as measured using the R-U5/pre-Gag (Long LTR) assay, which is present in only one copy per intact provirus. Finally, we calculated the ratios of one HIV transcript to another, which are independent of normalization to cell numbers and can be used to measure the presence and degree of different HIV transcriptional blocks [27].

### Collagenase treatment of PBMCs

HIV-infected, ART-suppressed individuals were recruited from the SCOPE cohort (individual 2475) and the VA Healthcare System (individual 129). Following a blood draw, PBMCs were isolated using Ficoll as previously described [26]. For individual 2475, a proportion of PBMCs (40x10^6^ cells) were untreated. The remaining cells were split for two treatment protocols: 1) 40x10^6^ PBMCs were treated with collagenase as described previously [26] and stained using the same antibodies employed for sorting CD4+ T cells from blood and gut (CD45, CD3, CD8, and LIVE/DEAD); 2) 40x10^6^ PBMCs were not treated with collagenase but stained using CD45, CD3, CD8, and LIVE/DEAD. A similar procedure was followed for individual 129, where 40x10^6^ PBMCs were untreated and 40x10^6^ cells were treated with collagenase, except these cells were not stained with fluorescently-conjugated antibodies. Following treatments, cells were counted and aliquoted into 10x10^6^ cells prior to centrifugation to pellet. After removal of supernatant, cells were directly lysed in TRI reagent and stored at -80°C until RNA and DNA extraction.

### Nucleic acid extraction

Total cellular RNA and DNA were extracted using TRI Reagent (Molecular Research Center, Inc., Cincinnati, OH) as per manufacturer’s instructions, with the following modifications: polyacryl carrier (Molecular Research Center, Inc., Cincinnati, OH) was added to TRI reagent prior to lysis, RNA was resuspended in RNase free-water, DNA was extracted using back extraction buffer (4M guanidine thiocyanate, 50mM sodium citrate, 1M Tris), polyacryl carrier was added to the aqueous phase containing the DNA, and DNA was resuspended in QIAGEN buffer EB.

### Reverse transcription

A common RT reaction was used to generate cDNA for all ddPCR assays except TAR [27]. Each 50μL RT contained cellular RNA, 5μL of 10x Superscript III buffer (Invitrogen, Carlsbad, CA), 5μL of 50mM MgCl_2_, 2.5μL of 50ng/μl random hexamers (Invitrogen), 2.5μL of 50μM dT15, 2.5μL of 10mM dNTPs, 1.25μL of 40U/μL RNaseOUT (Invitrogen), and 2.5μL of 200U/μL Superscript III RT (Invitrogen). Control RT reactions were performed in parallel with participant samples. A ‘6-assay’ synthetic HIV standard was utilized as a positive control [27]. HIV- donor PBMCs and water that was subjected to nucleic extraction by TRI Reagent served as negative controls for each transcript. The thermocycling conditions were as follows: 25.0°C for 10min, 50.0°C for 50min, followed by an inactivation step at 85.0°C for 5min.

### Polyadenylation-reverse transcription (polyA-RT) for TAR assay

Reverse transcription from a linker molecule (which we achieve using polyA polymerase to attach a polyA tail) is necessary for efficient reverse transcription of short, prematurely-terminated HIV transcripts limited to the TAR loop [31]. Therefore, a polyadenylation step was employed prior to reverse transcription and ddPCR for the TAR region [27, 31]. Each polyadenylation reaction comprised cellular RNA with 3μL of 10x Superscript III buffer (Invitrogen), 3μL of 50mM MgCl_2_, 1μL of 10mM ATP (Epicentre), 2μL of 4U/μL poly-A polymerase (Epicentre), and 1μL of 40U/μL RNaseOUT (Invitrogen) in a 20μL reaction. The reaction was incubated at 37μC for 45min prior to addition of RT reaction components, including 1.5μL of 10mM dNTPs (Invitrogen), 1.5μL of 50ng/μL random hexamers (Invitrogen), 1.5μL of 50μM oligo dT15, and 1μL of 200U/μL Superscript III reverse transcriptase (Invitrogen). Reverse transcription was performed on the final 30μL reaction at 25.0°C for 10 min, 50.0°C for 50 min, followed by an inactivation step at 85.0°C for 5 min.

### Droplet digital PCR (ddPCR)

Droplet digital PCR was employed because it enables absolute quantification, circumvents the requirement for external HIV standards, and is more forgiving of differences in PCR efficiency due to sequence mismatches [90]. These assays have been validated previously [27, 31]. cDNA from each sample was assayed in duplicate wells for Read-through, TAR, Long LTR, PolyA, and MS Tat-Rev regions (all samples) and (for rectal biopsies and PBMCs) one replicate for Nef. Total cellular DNA was used for the following ddPCR assays: 1) TERT (all samples); 2) Long LTR DNA (all samples); 3) U3-U5 ("Read-through"), TAR, and Nef DNA (PBMCs and rectal biopsies); and 4) 2-LTR circles (sorted CD4+ T cells). Each reaction consisted of 20μL containing cDNA (5μL) or DNA, 10μL of ddPCR Supermix for Probes (no dUTP) (Bio-Rad, Hercules, CA), 900 nM of primers, and 250 nM of probe. Following production of droplet emulsions using the QX100 Droplet Generator (Bio-Rad), the samples were amplified under the following thermocycling conditions: 10 minutes at 95°C, 45 cycles of 30 seconds at 95°C and 59°C for 60 seconds, and a final droplet cure step of 10 minutes at 98°C, using a 7900 Thermal Cycler (Life Technologies, Carlsbad, CA). Droplets were quantified using the QX100 Droplet Reader (Bio-Rad Laboratories Inc., Hercules, CA) and analyzed using the QuantaSoft software (version 1.6.6, Bio-Rad Laboratories Inc., Hercules, CA) in the “Absolute” quantification mode. Gates were set above the negative controls. False positives were rare, generally limited to a single droplet, and usually identifiable by abnormally high fluorescence in both channels.

### ddPCR for 2-LTR circles

A new ddPCR assay was used to measure levels of 2-LTR circles. Primers were "F Kumar" (5’ GCCTCAATAAAGCTTGCCTTGA 3’; HXB2 522–543) and "R Butler mod 2-LTR" (5' YCCACAGATCAAGGATMTCTTGTC 3’; 51–28). The probe, "P Kumar" (5’ CCAGAGTCACACAACAGACGGGCACA 3’; 559–84, was dual labelled with FAM (5') and Black Hole Quencher (BHQ; 3'). Each reaction consisted of 20μL containing DNA, 10μL of ddPCR Supermix for Probes (Bio-Rad, Hercules, CA), 900 nM of primers, and 250 nM of probe. Thermocycling conditions and analysis were as described above. A 2-LTR junction standard was created to determine the performance characteristics of the assay. The 2-LTR junction region was amplified from HIV NL4-3 infected PBMCs using the primers "F Buzon mod 2-LTR" (5’ CTARCTAGGGAACCCACTGCT 3’; HXB-2 498–518) and "R Buzon 2-LTR" (5’ GTAGTTCTGCCAATCAGGGAAG 3’; 92–71), then cloned and sequenced. The copy numbers in the standard were determined using the calculated molecular weight and the DNA concentration as determined by NanoDrop. Replicate dilutions of the standard were used in replicate experiments to determine the detection limit, efficiency, and linearity (S3 Fig). Detection frequencies were 2/6 at 0.9 copy, 3/6 at 1.8 copies, and 6/6 at 3.6 copies or above. No apparent inhibition was observed with addition of 1 or 2μg of QIAshredded cellular DNA.

### Statistics

The Wilcoxon signed rank test was performed to assess differences between levels of different HIV RNA or DNA sequence regions. Wells with no positive droplets (most common for MS Tat Rev) were assigned a value of zero for purposes of calculating the median and p values in Figs 2 and 4, and S1 Fig. The Wilcoxon signed rank tests were also repeated with no value (blank) for the undetectables, which did not change the major findings. GraphPad Prism (Version 5.0) was used for the Wilcoxon tests and exponential one phase decay modeling. As an additional method to account for undetectable samples, a negative binomial regression analysis was performed in STATA using the cell equivalents used in each ddPCR well and the number of replicates. The major findings did not change.

## Supporting information

S1 Table Half-lives for HIV transcripts in peripheral CD4+ T cells from an ART-suppressed individual *ex vivo*. (PDF)

S2 Table Sorted CD4+ T cell numbers for blood, rectum and ileum. (PDF)

S1 Fig Copies of HIV DNA per million cells in PBMCs and intact rectal biopsies. HIV DNA copies per million cells (normalized by reference gene, TERT) from (A) cryopreserved PBMCs, and (B) intact rectal biopsies are shown (*n = 9 matched donors*). HIV DNA copies were measured using the same primers/probes and ddPCR conditions used to measure levels of each HIV RNA. The median is represented by the black bar. Comparisons between DNA copies were performed using the Wilcoxon signed-rank test.
(TIF)

S2 Fig RNA copies per million cells in peripheral CD4+ T cells from an ART-suppressed individual *ex vivo*. CD4+ T cells were culture in the absence of RNA Pol II inhibitors and treated with 0.1% DMSO as vehicle control. Total RNA was extracted from cells harvested at three timepoints (0, 1 and 16 hours). Levels of each HIV transcripts were measured using RT-ddPCR.
(TIF)

S3 Fig Efficiency for HIV 2-LTR circle standard. To assess the efficiency of our HIV 2-LTR circle assay, the copy numbers in the 2-LTR standard were determined using the calculated molecular weight and the DNA concentration as determined by NanoDrop. Replicate dilutions of the standard were used in replicate experiments to determine the detection limit, efficiency, and linearity.
(TIF)

S4 Fig HIV cDNA copies per ddPCR well for sorted CD4+ T cells. Replicate values for each HIV transcript (expressed as absolute copies per ddPCR well) are shown for each individual. Total cell number analyzed is reported in parentheses for each anatomic site. Note that these absolute copy numbers per ddPCR well are not corrected for the RNA input into the reverse transcription reaction or the fraction of the reverse transcription that is used for each ddPCR well, which differ between TAR and the other assays.
(TIF)

S5 Fig HIV RNA/DNA ratios for peripheral, rectal, and ileal CD4+ T cells. The levels of (A) HIV RNA, and (B) average levels per provirus of each transcript (ratio of each HIV RNA to the Long LTR HIV DNA) were measured in CD4+ T cells isolated from the blood, rectum and ileum *(n = 4 matched individuals)*.
(TIF)

S6 Fig Effect of FACS-staining and collagenase on HIV transcription. The levels of HIV RNAs were assessed in (A) PBMCs with: 1) no further processing; 2) staining for FACS; and 3) collagenase treatment and FACS staining; and (B) PBMCs with and without collagenase treatment (no FACS staining). Data are reported as copies per million cells normalized by RNA mass or TERT, and copies per provirus.
(TIF)
