# Supplementary material for: Gut and blood differ in constitutive blocks to HIV transcription, suggesting tissue-specific differences in the mechanisms that govern HIV latency
Source: PLoS Pathog. 2018 Nov 15;14(11):e1007357. doi: 10.1371/journal.ppat.1007357 (PMC6237391; doi:10.1371/journal.ppat.1007357)
Supplement: S1 Table — (PDF) [file ppat.1007357.s001.pdf]

S1 Table. Half-lives for HIV transcripts in peripheral CD4+ T cells from an ART-suppressed individual *ex vivo*.

	**Triptolide**						**Actinomycin D**					
Normalised by	***DNA mass***		***RNA Mass***		***Cell count***		***DNA mass***		***RNA Mass***		***Cell count***	
	**$T_{(1/2)}$ [hours]**	**R^2**	**$T_{(1/2)}$ [hours]**	**R^2**	**$T_{(1/2)}$ [hours]**	**R^2**	**$T_{(1/2)}$ [hours]**	**R^2**	**$T_{(1/2)}$ [hours]**	**R^2**	**$T_{(1/2)}$ [hours]**	**R^2**
Readthrough	4.58	*0.99*	6.76	*0.97*	7.54	*0.95*	2.54	0.99	1.98	*0.99*	1.85	*0.99*
TAR	3.13	*0.99*	4.85	*0.99*	5.05	*0.97*	5.11	0.99	5.01	*0.99*	4.06	*0.99*
Long LTR	2.86	*0.99*	4.25	*0.98*	4.45	*0.93*	4.36	0.99	4.15	*0.99*	3.23	*0.98*
Nef	2.80	*0.99*	4.2	*0.99*	4.46	*0.93*	1.66	0.96	1.28	*0.96*	1.24	*0.96*
PolyA	2.29	*0.94*	3.67	*0.92*	3.87	*0.79*	2.34	0.97	1.80	*0.97*	1.69	*0.97*
MS Tat-Rev	6.25	*0.85*	13.96	*0.74*	16.95	*0.58*	5.49	0.92	4.81	*0.92*	4.37	*0.98*
