# Supplementary material for: Gut and blood differ in constitutive blocks to HIV transcription, suggesting tissue-specific differences in the mechanisms that govern HIV latency
Source: PLoS Pathog. 2018 Nov 15;14(11):e1007357. doi: 10.1371/journal.ppat.1007357 (PMC6237391; doi:10.1371/journal.ppat.1007357)
Supplement: S2 Table — (PDF) [file ppat.1007357.s002.pdf]

S2 Table. Cells isolated from each tissue, by individual

Sample ID	Site	Population	Sort Count
7244	Blood	CD4+T	$1.78 x 10^6$
7244	Rectum	CD4+T	$3.60 x 10^5$
7244	Ileum	CD4+T	$2.24 x 10^5$
2125	Blood	CD4+T	$2.49 x 10^6$
2125	Rectum	CD4+T	$7.26 x 10^5$
2475	Blood	CD4+T	$3.74 x 10^6$
2475	Rectum	CD4+T	$9.77 x 10^5$
2298	Blood	CD4+T	$2.25 x 10^6$
2298	Rectum	CD4+T	$6.59 x 10^5$
7253	Blood	CD4+T	$2.29 x 10^6$
7253	Ileum	CD4+T	$2.22 x 10^4$
7253	Rectum	CD4+T	$8.35 x 10^4$
7259	Blood	CD4+T	$3.00 x 10^6$
7259	Ileum	CD4+T	$3.80 x 10^4$
7259	Rectum	CD4+T	$2.31 x 10^5$
7260	Blood	CD4+T	$3.00 x 10^6$
7260	Ileum	CD4+T	$9.70 x 10^4$
7260	Rectum	CD4+T	$2.70 x 10^5$
