# Supplementary figures and images for: Gut and blood differ in constitutive blocks to HIV transcription, suggesting tissue-specific differences in the mechanisms that govern HIV latency
Source: PLoS Pathog. 2018 Nov 15;14(11):e1007357. doi: 10.1371/journal.ppat.1007357 (PMC6237391; doi:10.1371/journal.ppat.1007357)

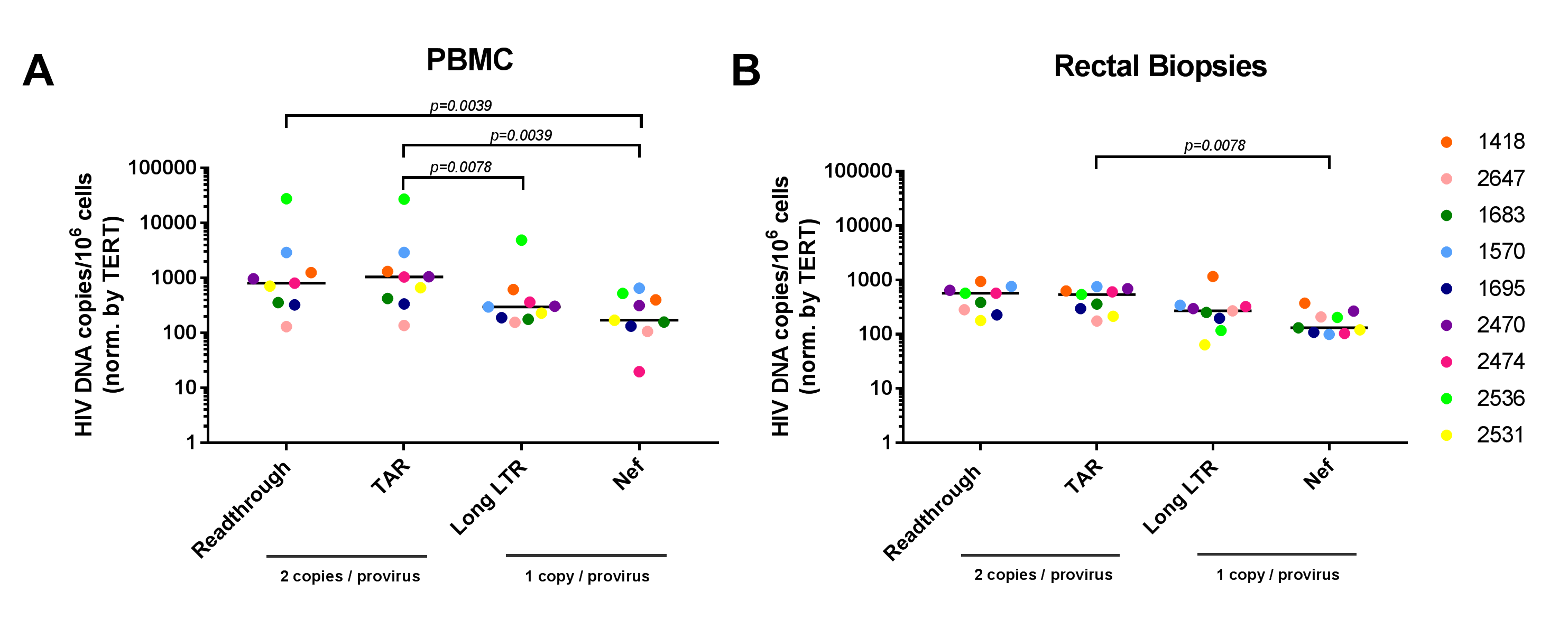

Supplement: S1 Fig — HIV DNA copies per million cells (normalized by reference gene, TERT) from (A) cryopreserved PBMCs, and (B) intact rectal biopsies are shown (n = 9 matched donors). HIV DNA copies were measured using the same primers/probes and ddPCR conditions used to measure levels of each HIV RNA. The median is represented by the black bar. Comparisons between DNA copies were performed using the Wilcoxon signed-rank test. (TIF) [file ppat.1007357.s003.tif]

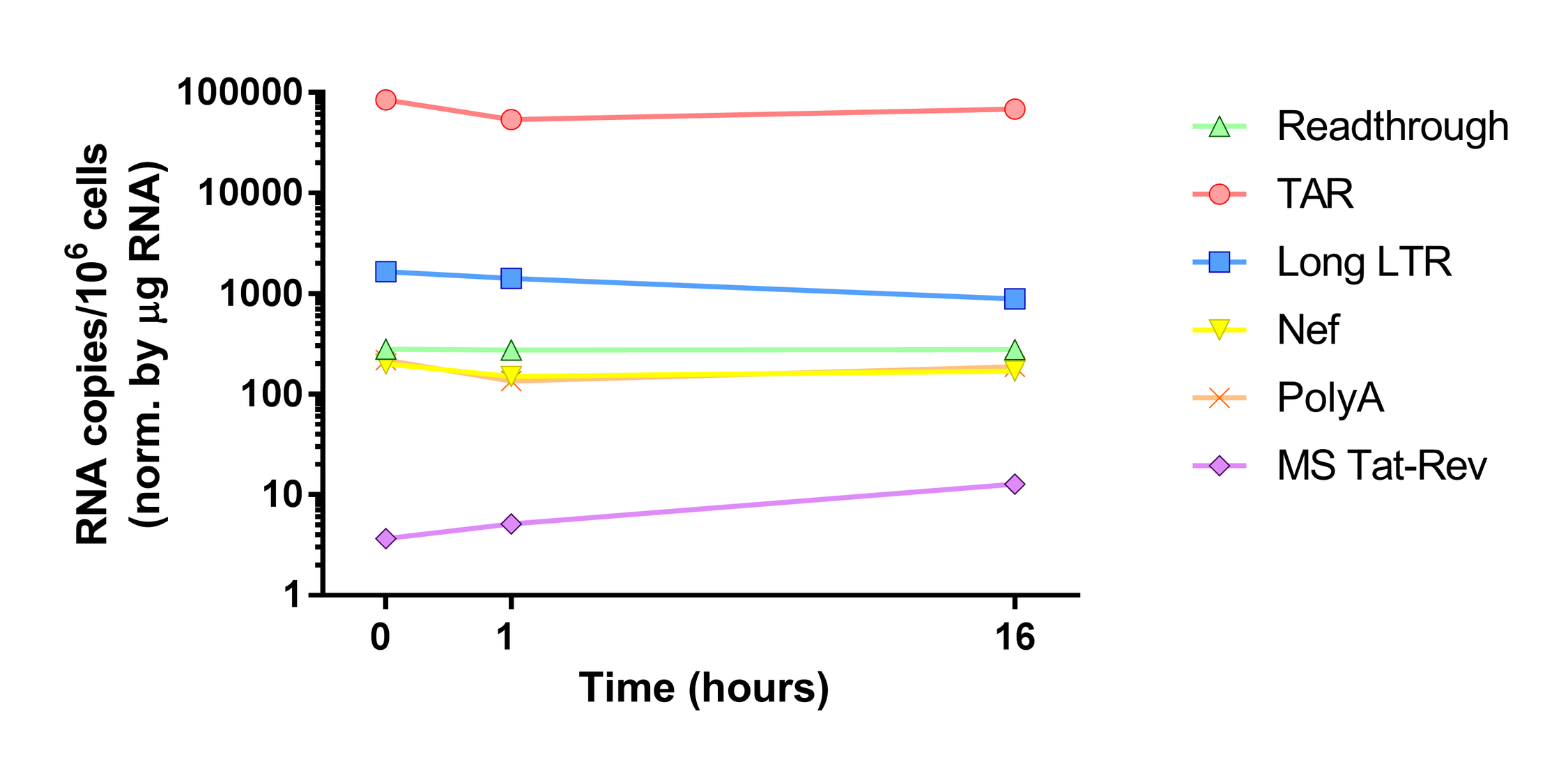

Supplement: S2 Fig — CD4+ T cells were culture in the absence of RNA Pol II inhibitors and treated with 0.1% DMSO as vehicle control. Total RNA was extracted from cells harvested at three timepoints (0, 1 and 16 hours). Levels of each HIV transcripts were measured using RT-ddPCR. (TIF) [file ppat.1007357.s004.tif]

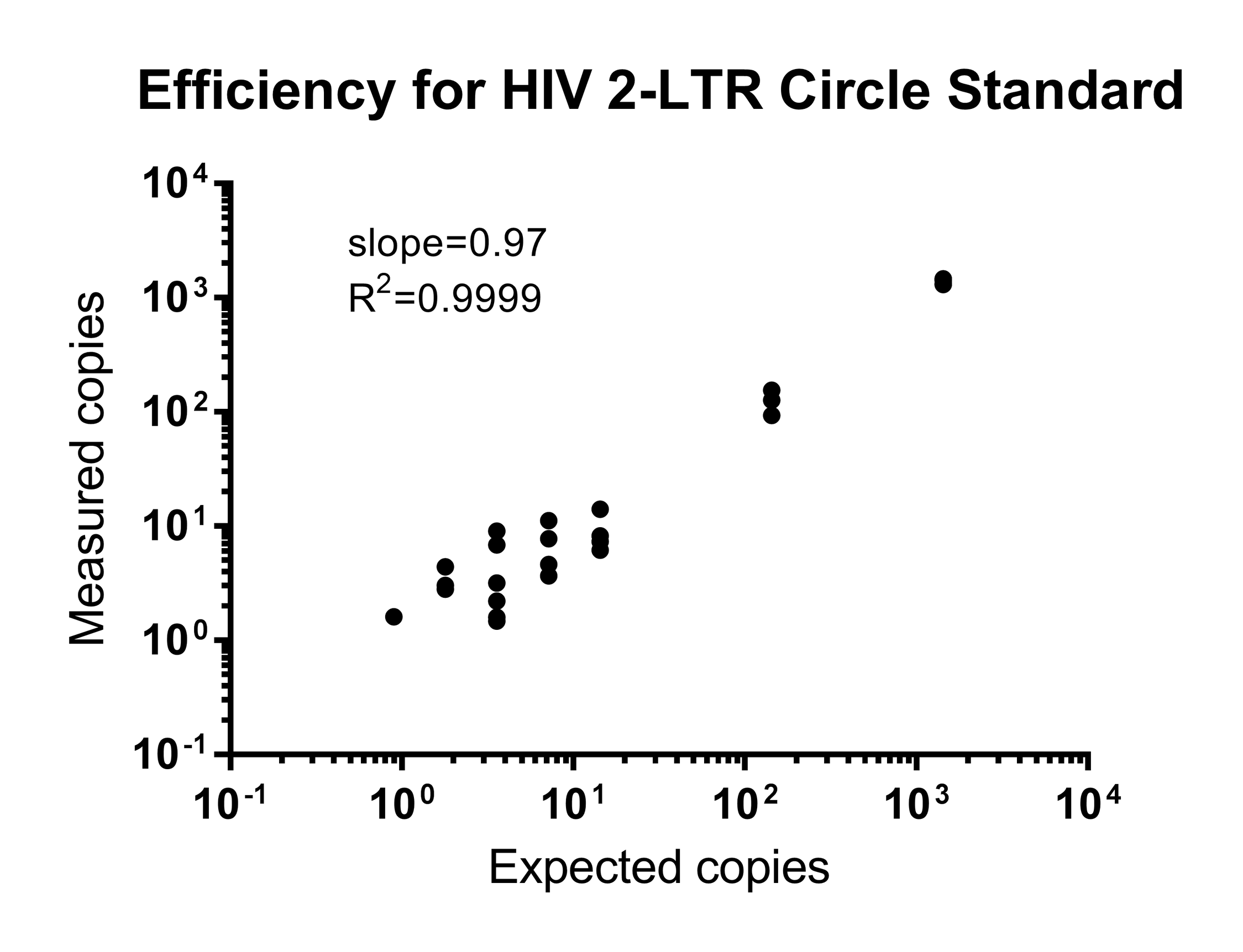

Supplement: S3 Fig — To assess the efficiency of our HIV 2-LTR circle assay, the copy numbers in the 2-LTR standard were determined using the calculated molecular weight and the DNA concentration as determined by NanoDrop. Replicate dilutions of the standard were used in replicate experiments to determine the detection limit, efficiency, and linearity. (TIF) [file ppat.1007357.s005.tif]

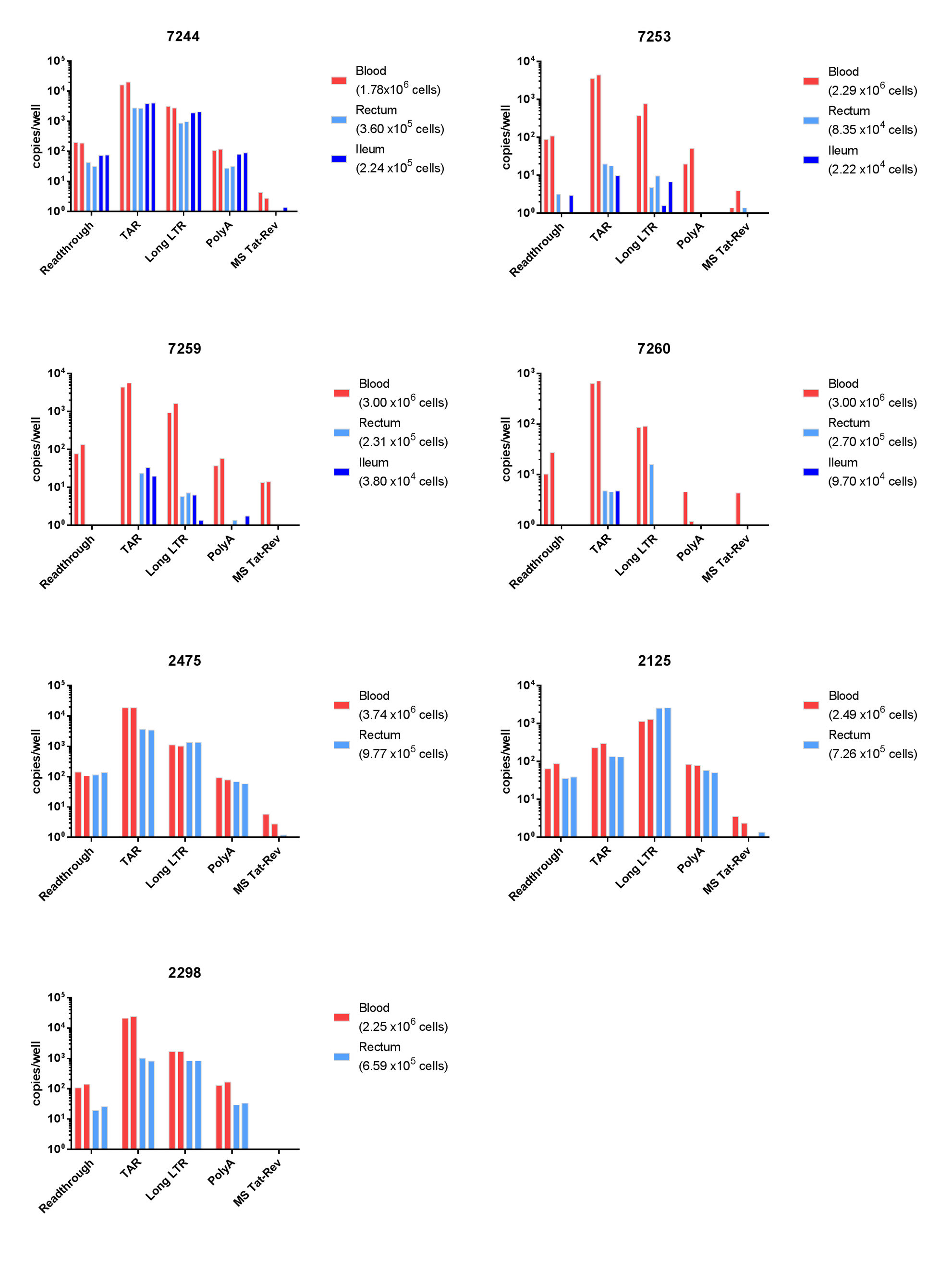

Supplement: S4 Fig — Replicate values for each HIV transcript (expressed as absolute copies per ddPCR well) are shown for each individual. Total cell number analyzed is reported in parentheses for each anatomic site. Note that these absolute copy numbers per ddPCR well are not corrected for the RNA input into the reverse transcription reaction or the fraction of the reverse transcription that is used for each ddPCR well, which differ between TAR and the other assays. (TIF) [file ppat.1007357.s006.tif]

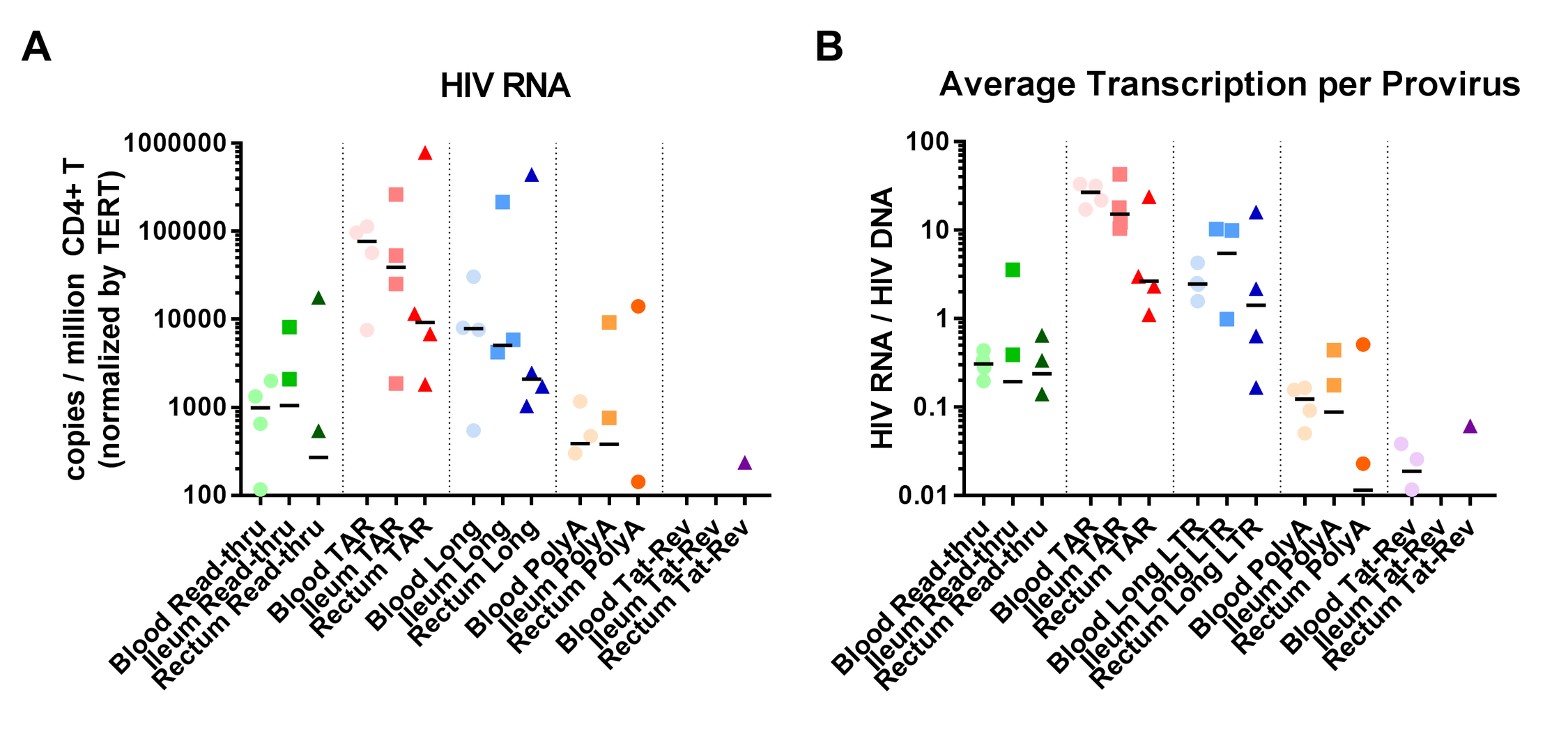

Supplement: S5 Fig — The levels of (A) HIV RNA, and (B) average levels per provirus of each transcript (ratio of each HIV RNA to the Long LTR HIV DNA) were measured in CD4+ T cells isolated from the blood, rectum and ileum (n = 4 matched individuals). (TIF) [file ppat.1007357.s007.tif]

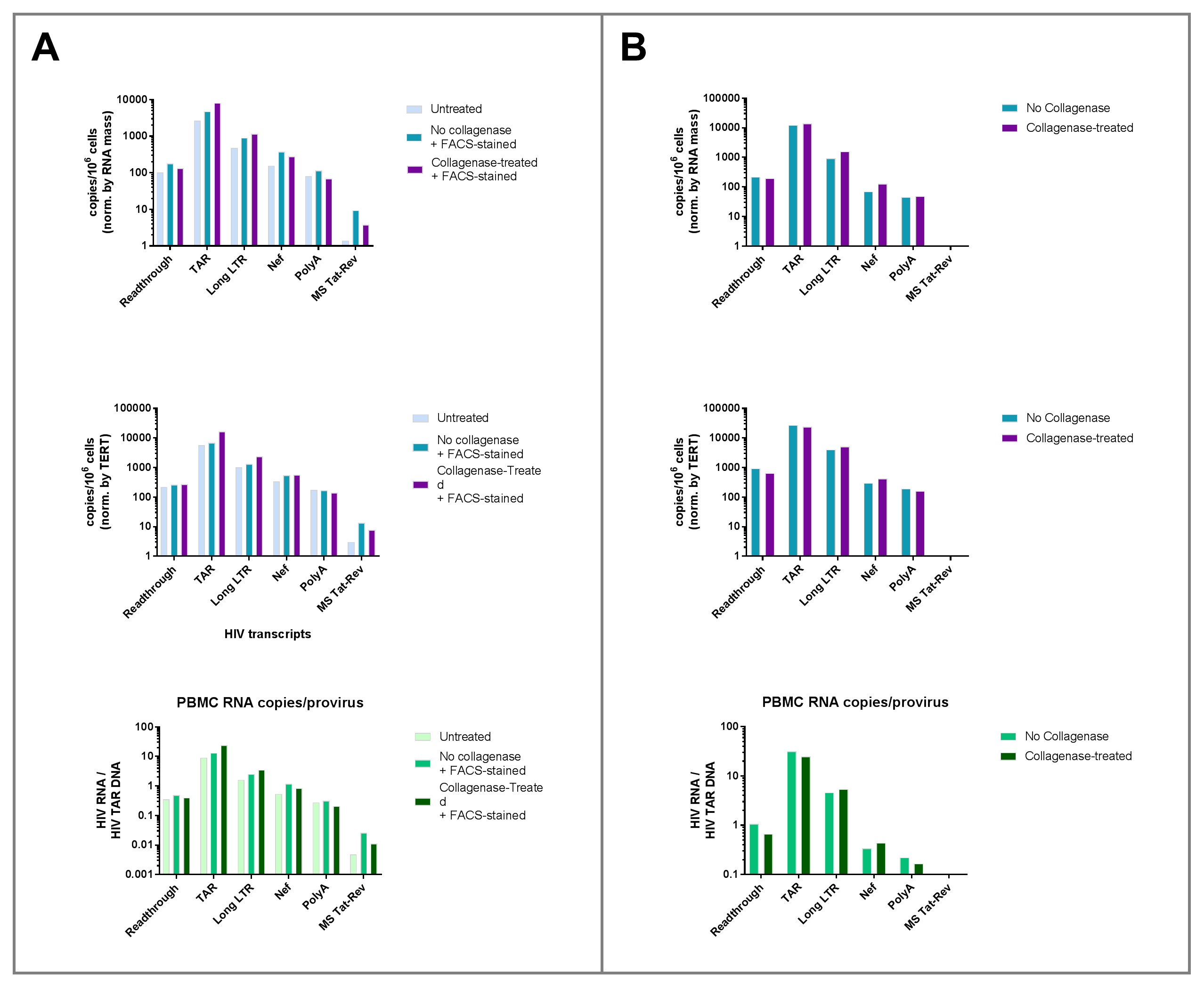

Supplement: S6 Fig — The levels of HIV RNAs were assessed in (A) PBMCs with: 1) no further processing; 2) staining for FACS; and 3) collagenase treatment and FACS staining; and (B) PBMCs with and without collagenase treatment (no FACS staining). Data are reported as copies per million cells normalized by RNA mass or TERT, and copies per provirus. (TIF) [file ppat.1007357.s008.tif]
